# Correction: Nonsense mutation suppression is enhanced by targeting different stages of the protein synthesis process

**DOI:** 10.1371/journal.pbio.3002524

**Published:** 2024-02-14

**Authors:** Amnon Wittenstein, Michal Caspi, Ido Rippin, Orna Elroy-Stein, Hagit Eldar-Finkelman, Sven Thoms, Rina Rosin-Arbesfeld

Fig 1 is erroneously duplicated as Fig 5. Please see the correct Fig 5 here.

**Fig 5:**
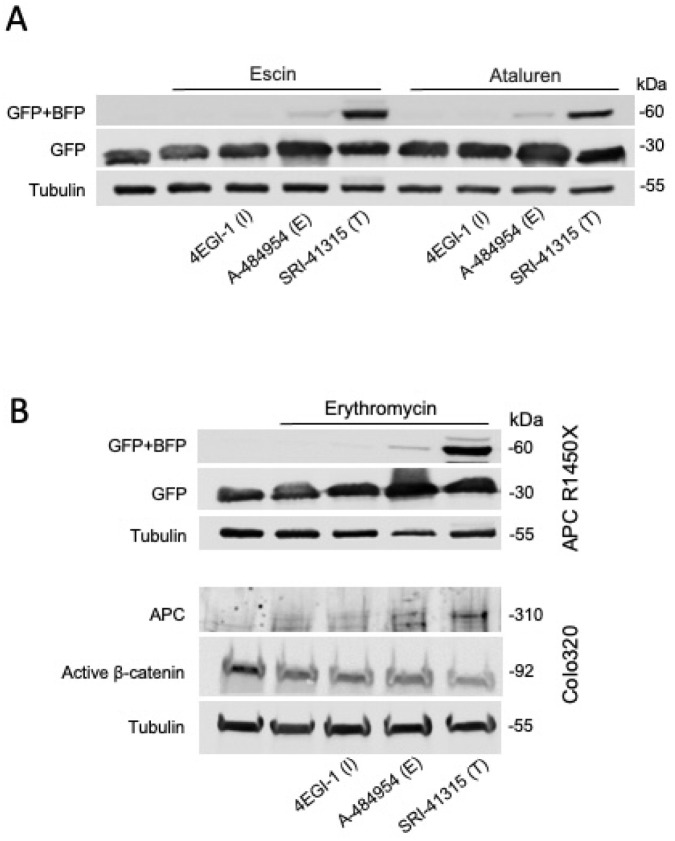
Targeting different stages of the protein synthesis process enhances readthrough mediated by non-aminoglycosides. **(A)** The APC R1450X reporter cell line was treated for 24 h with 10 μm Escin or 200 μm Ataluren and 50 μm 4EGI-1, 100 μm A-484954 or 5 μm SRI-41315. **(B)** The APC R1450X reporter cell line (upper blot) and Colo320 cell line (lower blot) were treated for 24 h with 300 μg/ml Erythromycin and 50 μm 4EGI-1, 100 μm A-484954 or 5 μm SRI-41315. APC, adenomatous polyposis coli.
