# Supplementary material for: Nonsense mutation suppression is enhanced by targeting different stages of the protein synthesis process
Source: PLoS Biol. 2023 Nov 9;21(11):e3002355. doi: 10.1371/journal.pbio.3002355 (PMC10684085; doi:10.1371/journal.pbio.3002355)
Supplement: S1 Fig — (A) HCT116, SW48, and Colo320 cell lines were treated with 500 μg/ml G418 for 24 h followed by WB analysis using the indicated antibodies. (B) SW403 and LOVO cell lines were treated with 1.5 mg/ml G418 for 24 h followed by WB analysis using the indicated antibodies. FS = Frameshift. (C) Colo320 and SW480 cell lines were treated as in A, followed by WB analysis using antibodies specific for APC and tubulin. (PPTX) [file pbio.3002355.s001.pptx]

## Slide 1
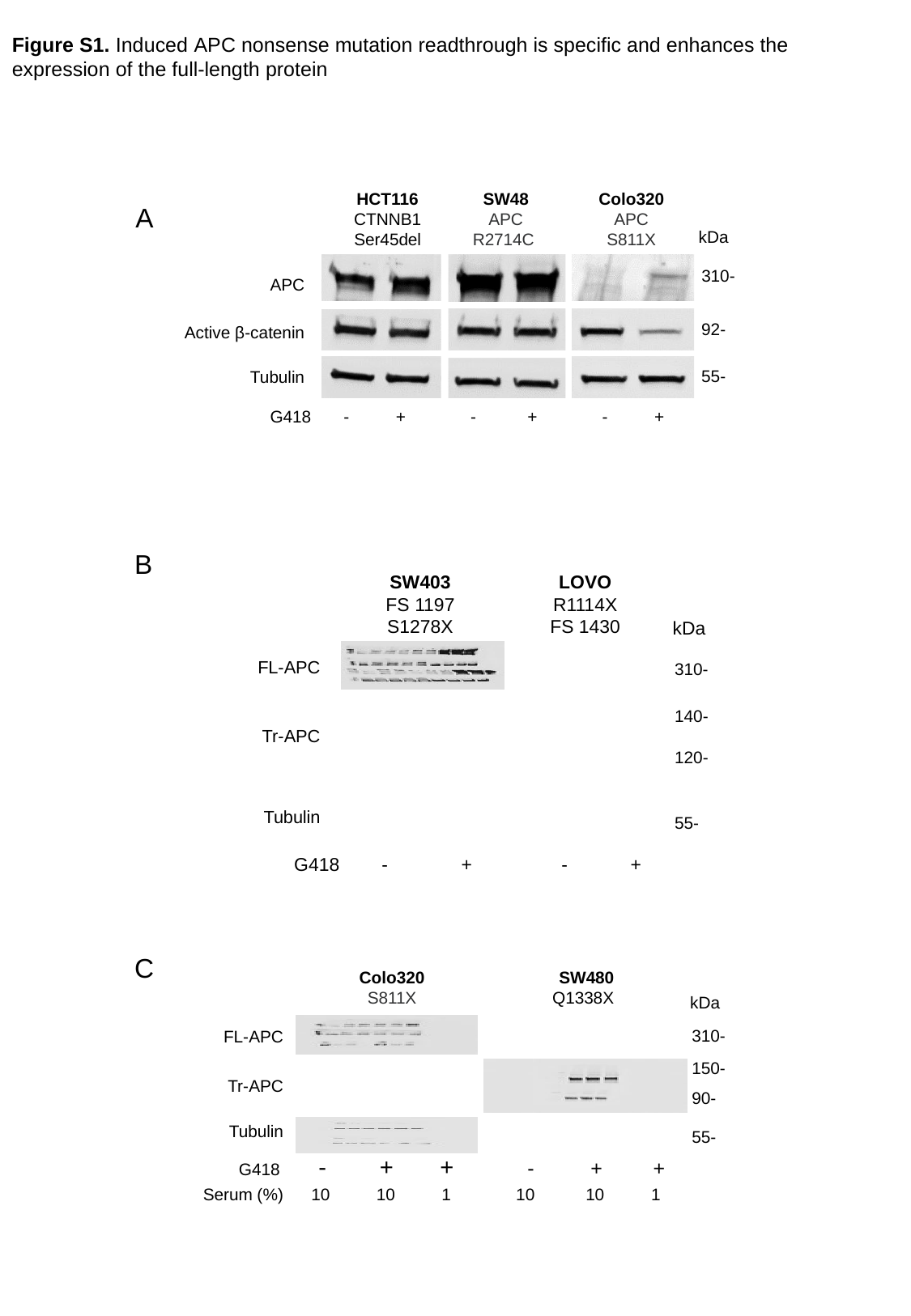

Figure S1. Induced APC nonsense mutation readthrough is specific and enhances the expression of the full-length protein
HCT116
CTNNB1 Ser45del
SW48
APC
 R2714C
Colo320
APC
S811X
A
kDa
-310
APC
-92
Active β-catenin
-55
Tubulin
G418 - + - + - +
B
SW403
1197 FS
S1278X
LOVO
R1114X
1430 FS
kDa
FL-APC
-310
-140
Tr-APC
-120
Tubulin
-55
G418 - + - +
C
Colo320
S811X
SW480
Q1338X
kDa
-310
FL-APC
-150
Tr-APC
-90
Tubulin
-55
G418 - + + - + +
Serum (%) 10 10 1 10 10 1
