# Supplementary material for: Nonsense mutation suppression is enhanced by targeting different stages of the protein synthesis process
Source: PLoS Biol. 2023 Nov 9;21(11):e3002355. doi: 10.1371/journal.pbio.3002355 (PMC10684085; doi:10.1371/journal.pbio.3002355)
Supplement: S2 Fig — (A) The APC R1450X reporter cell line was treated for 24 h with 500 μg/ml GM and/or 500 nM Torin-1 followed by WB. The graphs show the relative GFP-BFP band intensity (normalized to GFP band intensity). Bars represent the mean values ± SD from 4 independent experiments. P < 0.0001. (B, C) Colo320 (B) and SW403 (C) were treated for 24 h with 500 μg/ml G418 and/or 500 nM Torin-1 followed by WB analysis. Graphs represent the intensities of the APC/tubulin or active β-catenin/tubulin bands (arbitrary units), calculated by the Fusion-Capt analysis software. The bars represent the mean values ± SD from 2–4 independent experiments. Colo320: P < 0.0001, SW403: APC P = 0.0023, Active β-catenin P < 0.0001. Tukey’s multiple comparisons scores are shown. The data underlying the graphs in the figure can be found in S1 Data. (PPTX) [file pbio.3002355.s002.pptx]

## Slide 1
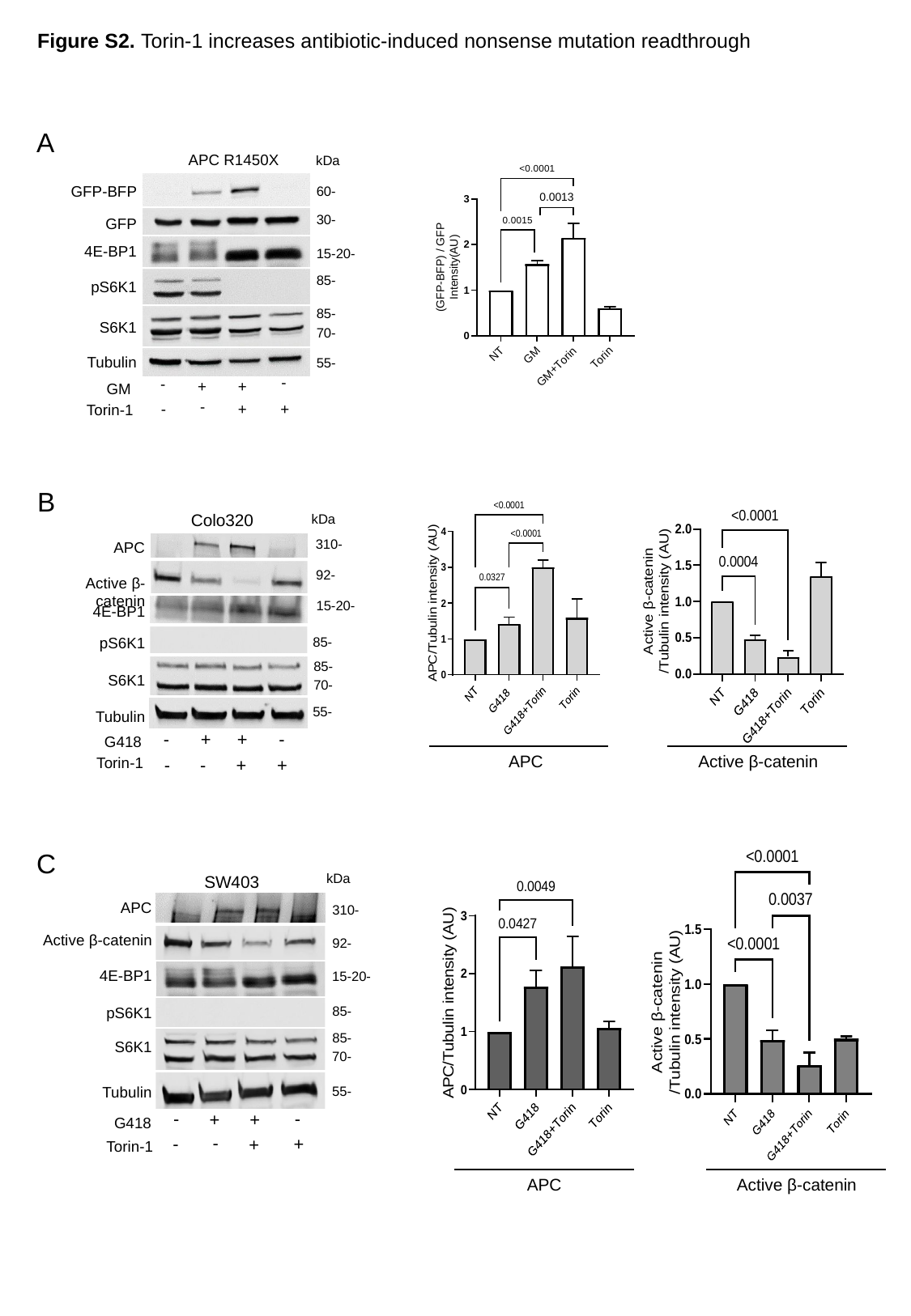

Figure S2. Torin-1 increases antibiotic-induced nonsense mutation readthrough
A
APC R1450X
kDa
GFP-BFP
-60
-30
GFP
4E-BP1
-15-20
-85
pS6K1
-85
S6K1
-70
Tubulin
-55
-
-
+
+
GM
-
-
+
+
Torin-1
B
Colo320
kDa
-310
APC
-92
Active β-catenin
-15-20
4E-BP1
pS6K1
-85
-85
S6K1
-70
-55
Tubulin
-
+
+
-
G418
APC
Active β-catenin
Torin-1
-
-
+
+
C
 SW403
kDa
APC
-310
Active β-catenin
-92
4E-BP1
-15-20
-85
pS6K1
-85
S6K1
-70
Tubulin
-55
-
-
+
+
G418
-
-
+
+
Torin-1
APC
Active β-catenin
