# Supplementary figures and images for: Nonsense mutation suppression is enhanced by targeting different stages of the protein synthesis process
Source: PLoS Biol. 2023 Nov 9;21(11):e3002355. doi: 10.1371/journal.pbio.3002355 (PMC10684085; doi:10.1371/journal.pbio.3002355)

## Slide 1
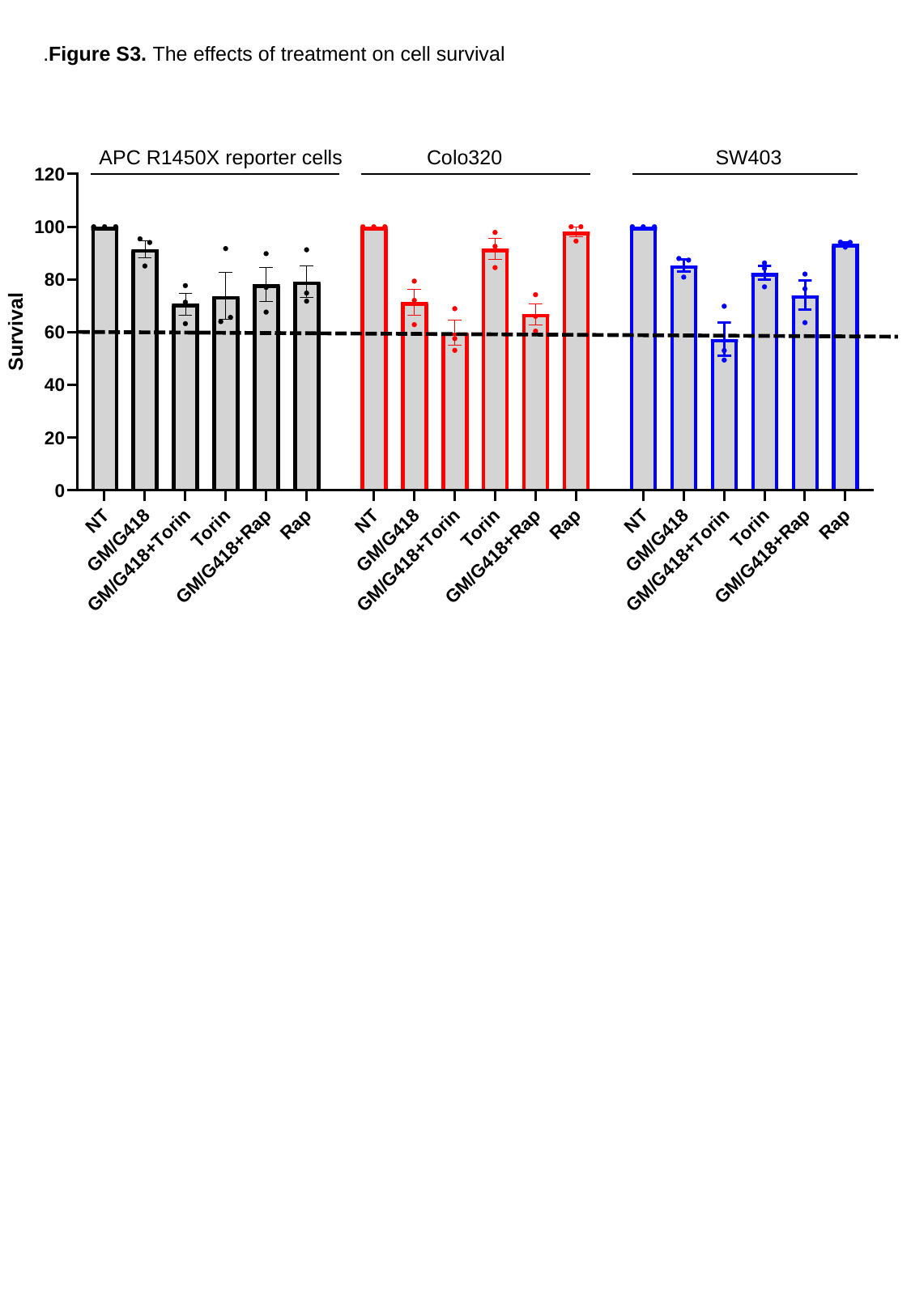

Figure S3. The effects of treatment on cell survival.
APC R1450X reporter cells
Colo320
SW403

Supplement: S3 Fig — The APC-1450X reporter cell line, Colo320 and SW403 cell lines were treated for 24 h with 500 μg/ml GM (APC-1450X reporter cell line) or G418 (Colo320 and SW403), 500 nM Torin-1 or 1 μm Rap. PrestoBlue reagent was added to the wells and absorbance was measured after 3 h incubation at 570 and 600 nm. The bars represent the mean values ± SD from 3 independent experiments for each treatment, compared to untreated cells in each cell line. The data underlying the graphs in the figure can be found in S1 Data. (PPTX) [file pbio.3002355.s003.pptx]

## Slide 1
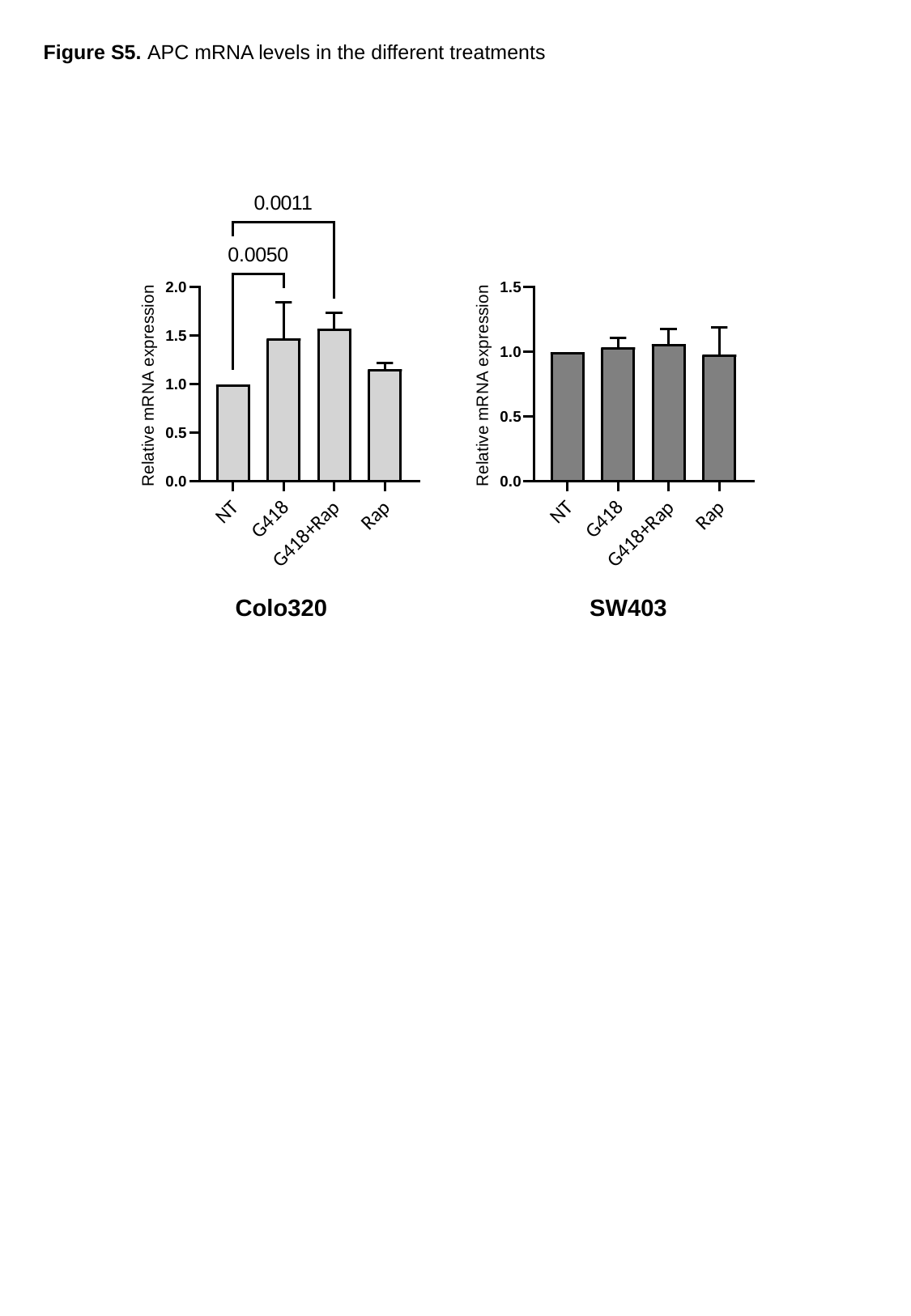

Figure S5. APC mRNA levels in the different treatments
Colo320
SW403

Supplement: S5 Fig — Colo320 and SW403 cell lines were treated for 24 h with 500 μg/ml G418 and/or 1 μm Rap. Total RNA was extracted from the treated samples, converted to cDNA, and subjected to RT-qPCR analysis. APC transcript levels were analyzed. The bars represent the mean values ± SD from 5 independent experiments. Two-way ANOVA (P = 0.0012) with Tukey’s multiple comparisons test was applied—significant scores were depicted. The data underlying the graphs in the figure can be found in S1 Data. (PPTX) [file pbio.3002355.s005.pptx]
