# Supplementary material for: Nonsense mutation suppression is enhanced by targeting different stages of the protein synthesis process
Source: PLoS Biol. 2023 Nov 9;21(11):e3002355. doi: 10.1371/journal.pbio.3002355 (PMC10684085; doi:10.1371/journal.pbio.3002355)
Supplement: S4 Fig — SW620 and SW837 were treated for 24 h (SW620) or 48 h (SW837) with 500 μg/ml G418 and/or 500 nM Torin-1 followed by WB analysis for the indicated proteins. (PPTX) [file pbio.3002355.s004.pptx]

## Slide 1
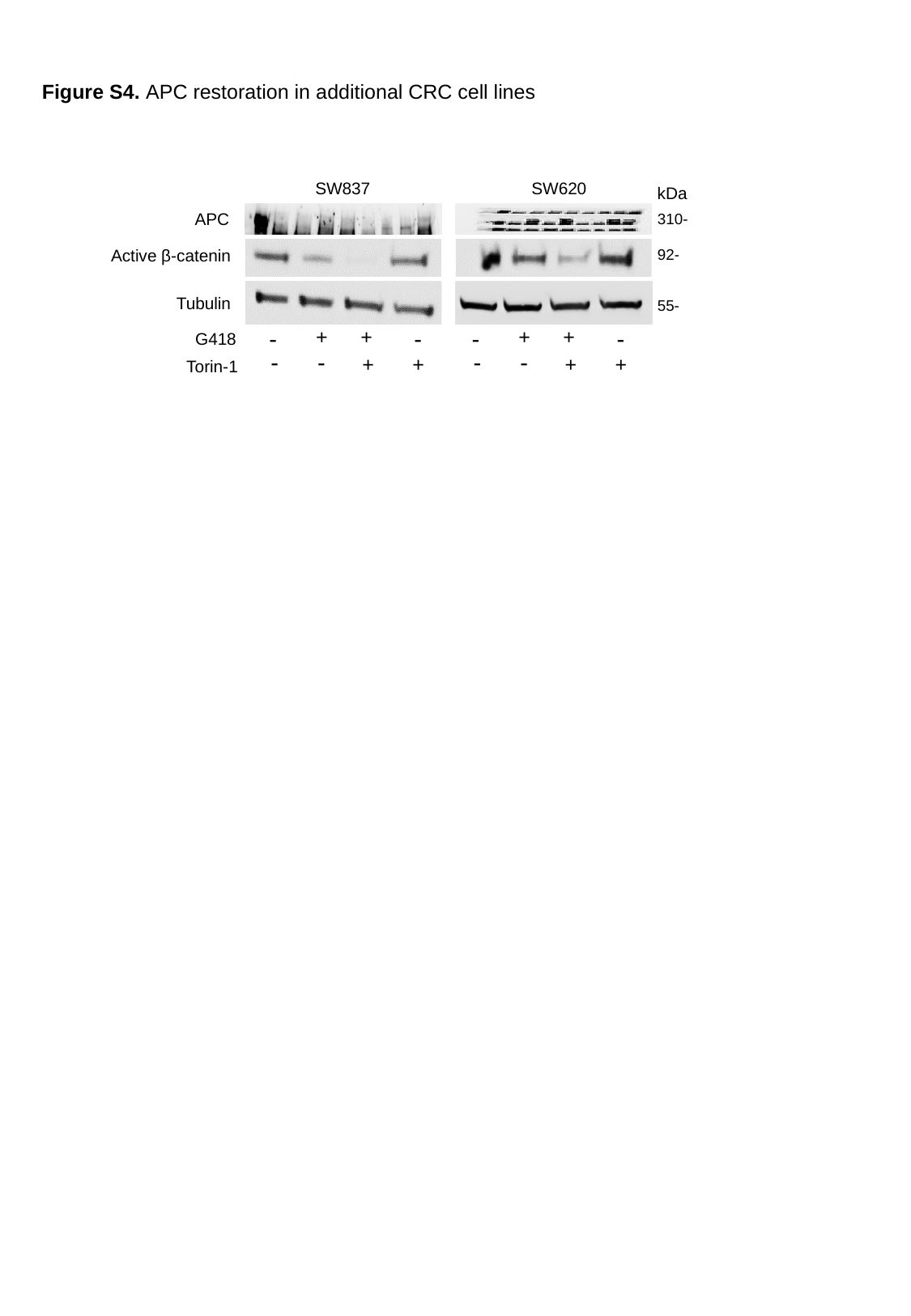

Figure S4. APC restoration in additional CRC cell lines
SW837
SW620
kDa
APC
-310
-92
Active β-catenin
Tubulin
-55
-
+
+
-
-
+
+
-
G418
-
-
-
-
+
+
+
+
Torin-1
