# Supplementary material for: Nonsense mutation suppression is enhanced by targeting different stages of the protein synthesis process
Source: PLoS Biol. 2023 Nov 9;21(11):e3002355. doi: 10.1371/journal.pbio.3002355 (PMC10684085; doi:10.1371/journal.pbio.3002355)
Supplement: S6 Fig — (A, B) Colo320 (A) and SW403 (B) cell lines were treated for 24 h with 500 μg/ml G418 or 50 μm 4EGI-1. The bars represent the mean values ± SD from 3–4 independent experiments. Colo320: P < 0.0001; SW403: active β-catenin P < 0.0001. Tukey’s multiple comparison scores are shown. (C, D) Colo320 (C) and SW403 (D) cell lines were treated for 24 h with 500 μg/ml G418 or 30 μm Tomivosertib. The bars represent the mean values ± SD from 3 independent experiments. Colo320: P = 0.0073; SW403: P = 0.0058. Tukey’s multiple comparison scores are shown. The data underlying the graphs in the figure can be found in S1 Data. (PPTX) [file pbio.3002355.s006.pptx]

## Slide 1
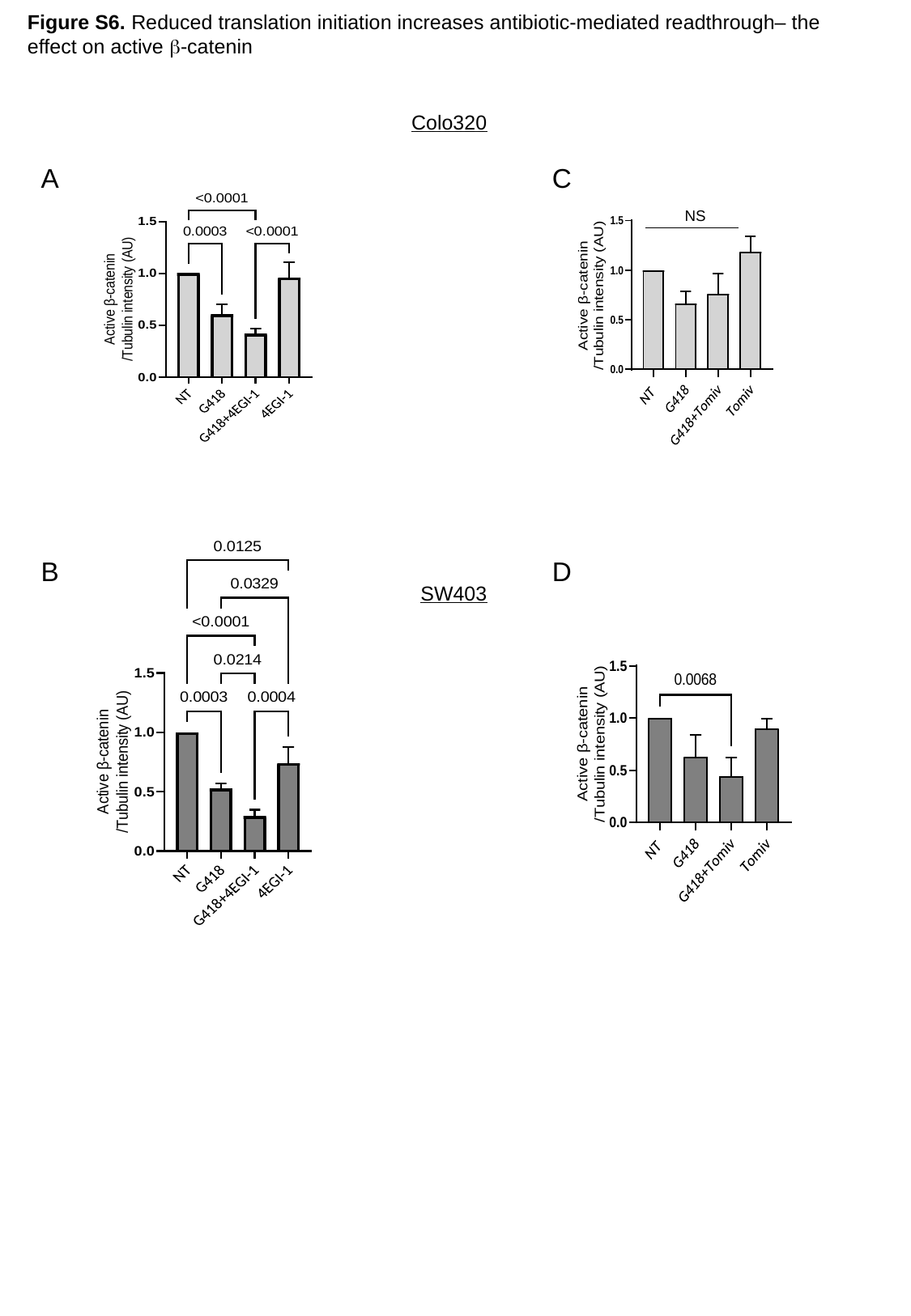

Figure S6. Reduced translation initiation increases antibiotic-mediated readthrough– the effect on active b-catenin
Colo320
A
C
NS
B
D
SW403
