# Supplementary material for: Nonsense mutation suppression is enhanced by targeting different stages of the protein synthesis process
Source: PLoS Biol. 2023 Nov 9;21(11):e3002355. doi: 10.1371/journal.pbio.3002355 (PMC10684085; doi:10.1371/journal.pbio.3002355)
Supplement: S7 Fig — The APC 1450X reporter cell line was treated for 24 h with 500 μg/ml GM and 0.5, 1, 5, 10, or 20 μm PF-4708671 (S6K1 inhibitor) followed by WB analysis using the indicated antibodies. The graphs represent the relative GFP-BFP band intensity (normalized to GFP band intensity, with or without 20 μm PF-4708671), calculated by the Fusion-Capt analysis software. The bars represent the mean values ± SD from 4 independent experiments. The data underlying the graphs in the figure can be found in S1 Data. (PPTX) [file pbio.3002355.s007.pptx]

## Slide 1
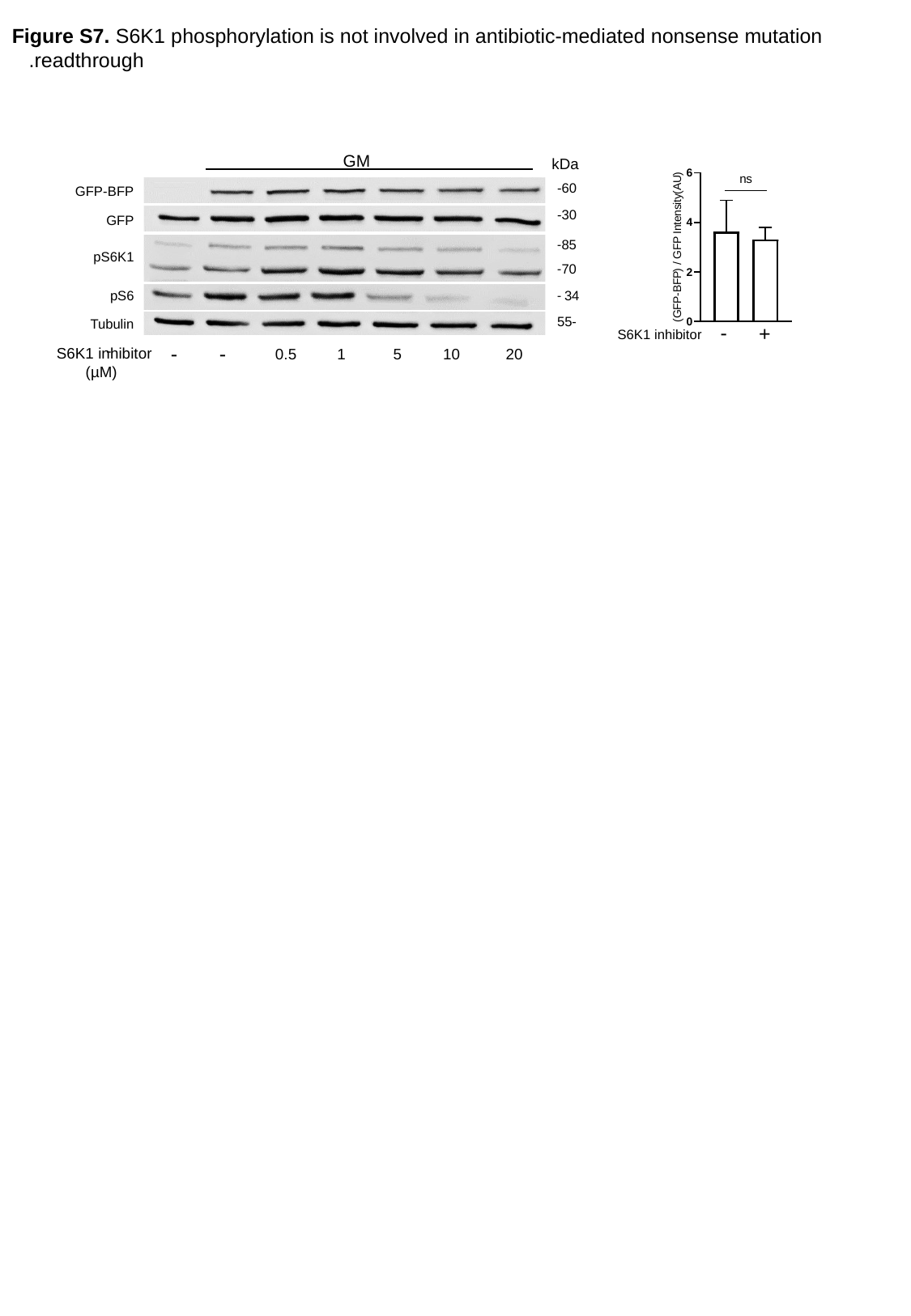

Figure S7. S6K1 phosphorylation is not involved in antibiotic-mediated nonsense mutation readthrough.
GM
kDa
-
+
S6K1 inhibitor
-60
GFP-BFP
-30
GFP
-85
pS6K1
-70
- 34
pS6
-55
Tubulin
-
-
-
S6K1 inhibitor
(µM)
20
0.5
1
5
10
