# Supplementary material for: Nonsense mutation suppression is enhanced by targeting different stages of the protein synthesis process
Source: PLoS Biol. 2023 Nov 9;21(11):e3002355. doi: 10.1371/journal.pbio.3002355 (PMC10684085; doi:10.1371/journal.pbio.3002355)
Supplement: S8 Fig — The APC 1450X reporter cell line was treated for 24 h with 500 μg/ml GM and 100 μg/ml Apidaecin or 500 μm N-Oxalylglycine (NOG). The bars represent the relative GFP-BFP band intensity (normalized to GFP band intensity) mean values ± SD from 3 independent experiments. P < 0.0001. The data underlying the graphs in the figure can be found in S1 Data. (PPTX) [file pbio.3002355.s008.pptx]

## Slide 1
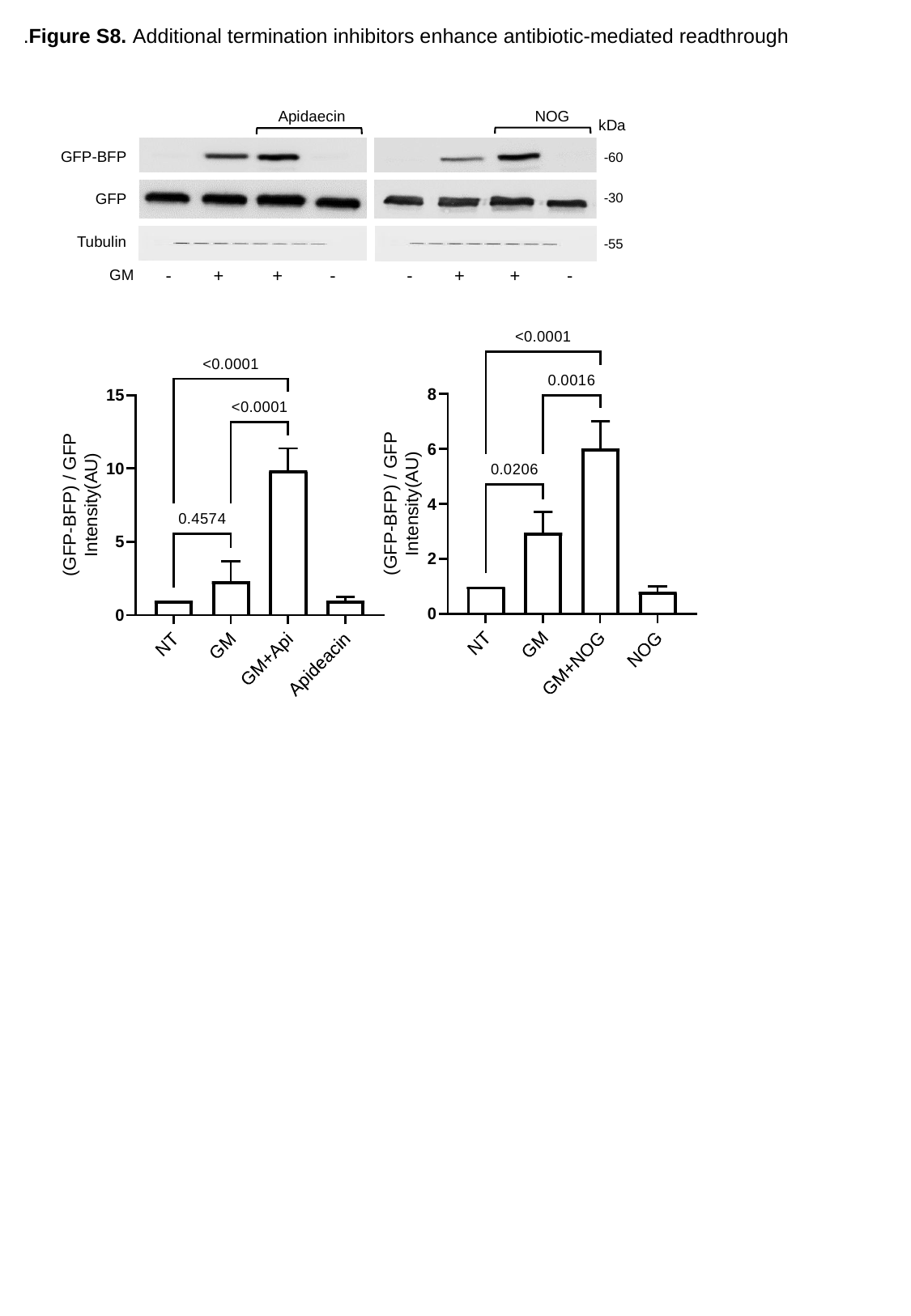

Figure S8. Additional termination inhibitors enhance antibiotic-mediated readthrough.
Apidaecin
NOG
kDa
GFP-BFP
-60
GFP
-30
Tubulin
-55
-
+
+
-
-
+
+
-
GM
