# Supplementary material for: Nonsense mutation suppression is enhanced by targeting different stages of the protein synthesis process
Source: PLoS Biol. 2023 Nov 9;21(11):e3002355. doi: 10.1371/journal.pbio.3002355 (PMC10684085; doi:10.1371/journal.pbio.3002355)

FIG1

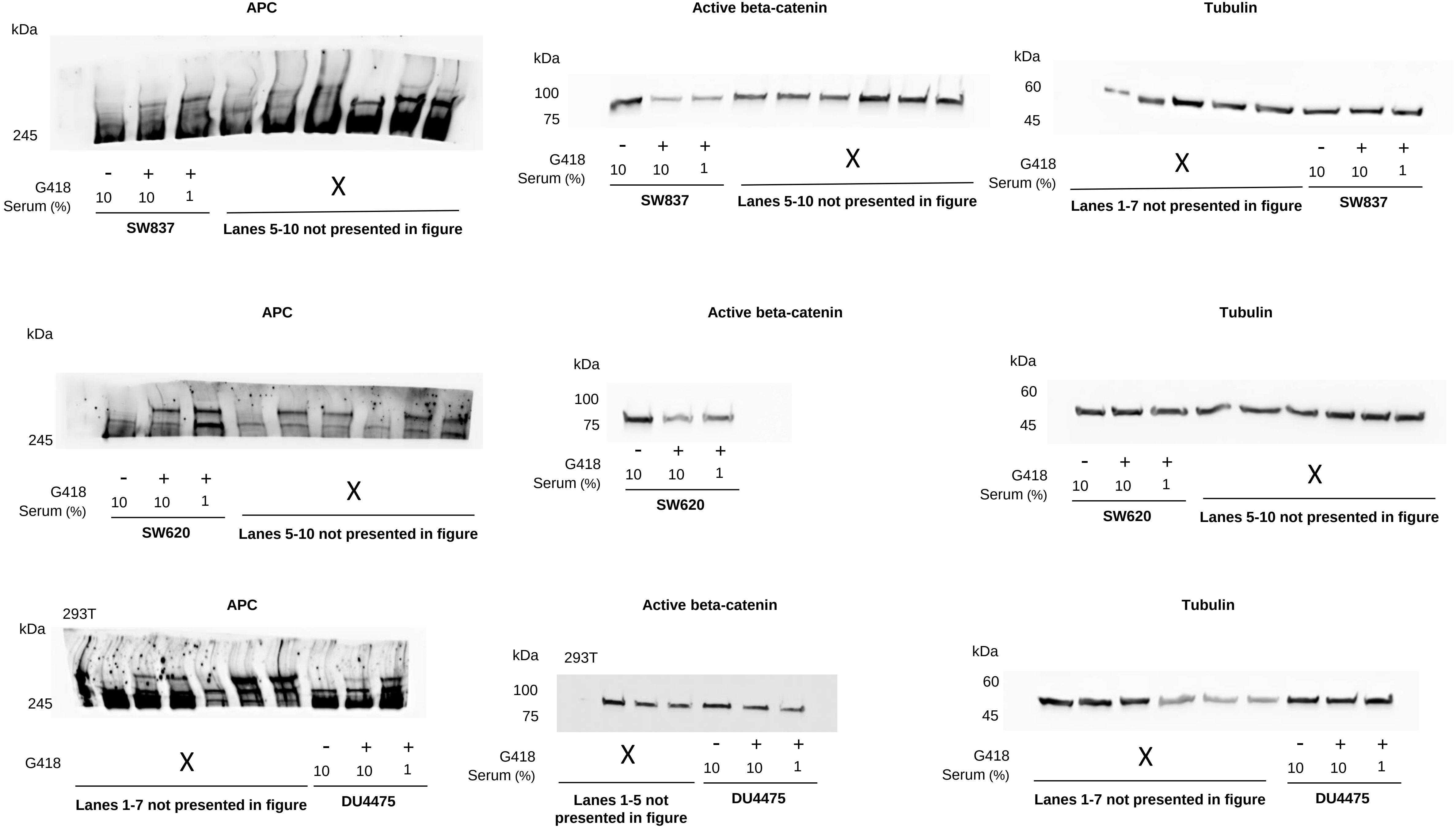
APC
kDa
245
G418 - + +
Serum (%) 10 10 1
SW837
X
Lanes 5-10 not presented in figure
Active beta-catenin
kDa
100
75
G418 - + +
Serum (%) 10 10 1
SW837
X
Lanes 5-10 not presented in figure
Tubulin
kDa
60
45
G418
Serum (%)
X
Lanes 1-7 not presented in figure
- + +
10 10 1
SW837
APC
kDa
245
G418 - + +
Serum (%) 10 10 1
SW620
X
Lanes 5-10 not presented in figure
Active beta-catenin
kDa
100
75
G418 - + +
Serum (%) 10 10 1
SW620
Tubulin
kDa
60
45
G418 - + +
Serum (%) 10 10 1
SW620
X
Lanes 5-10 not presented in figure
293T
APC
kDa
245
G418
X
Lanes 1-7 not presented in figure
- + +
10 10 1
DU4475
Active beta-catenin
kDa
293T
100
75
G418
Serum (%)
X
Lanes 1-5 not presented in figure
- + +
10 10 1
DU4475
Tubulin
kDa
60
45
G418
Serum (%)
X
Lanes 1-7 not presented in figure
- + +
10 10 1
DU4475

FIG1

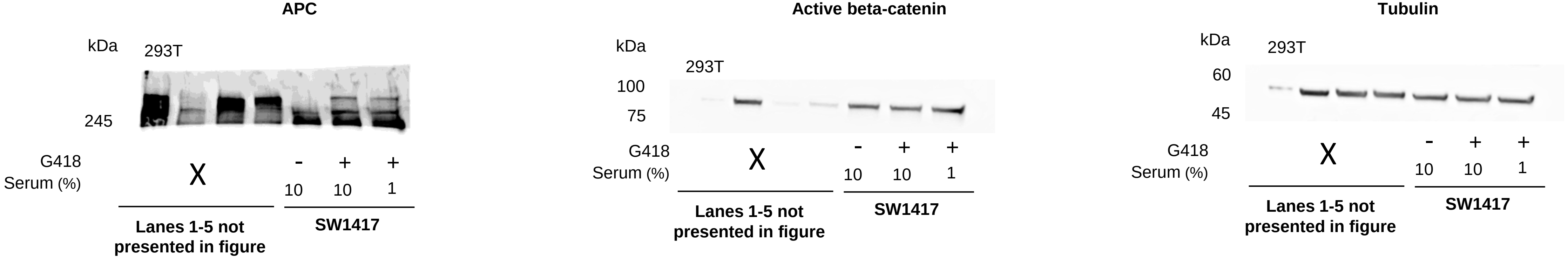
APC
kDa
293T
245
G418
Serum (%)
X
- + +
10 10 1
Lanes 1-5 not presented in figure
SW1417
Active beta-catenin
kDa
293T
100
75
G418
Serum (%)
X
- + +
10 10 1
Lanes 1-5 not presented in figure
SW1417
Tubulin
kDa
293T
60
45
G418
Serum (%)
X
- + +
10 10 1
Lanes 1-5 not presented in figure
SW1417

FIG2A

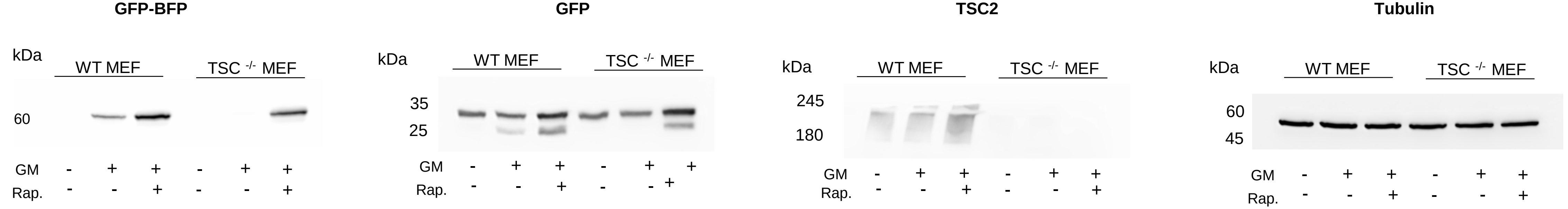

FIG2B

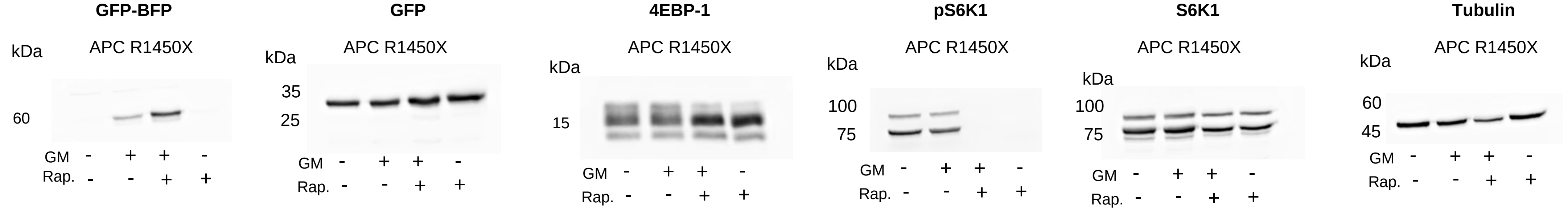

FIG2C

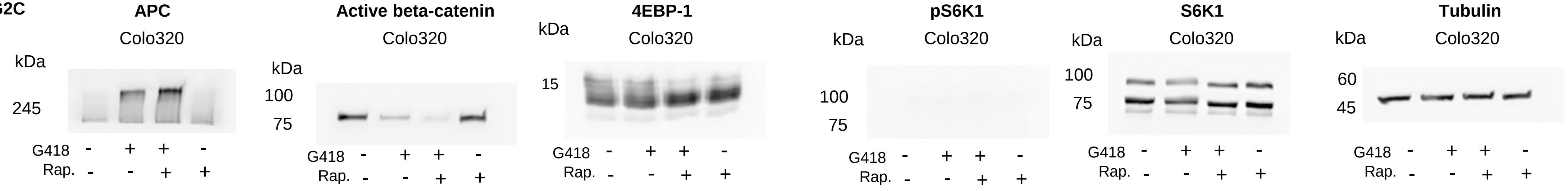

FIG2D

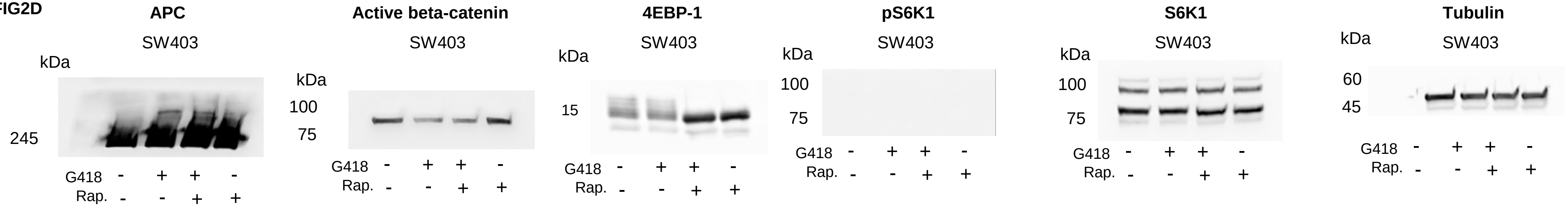

FIG3A

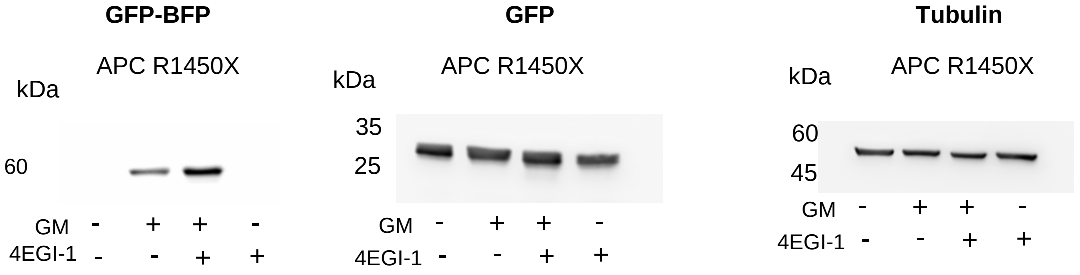

FIG3B

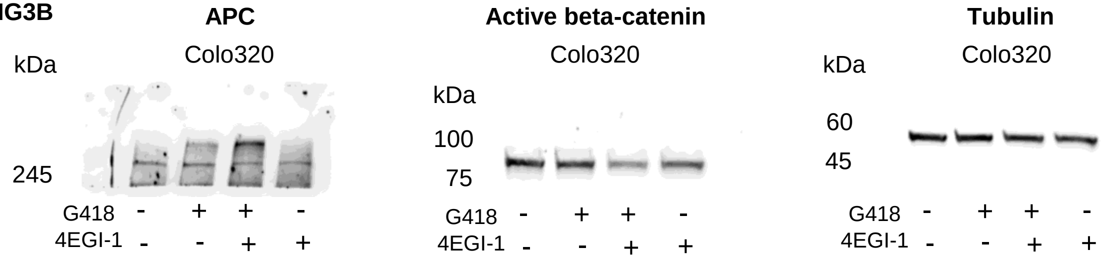

FIG3C

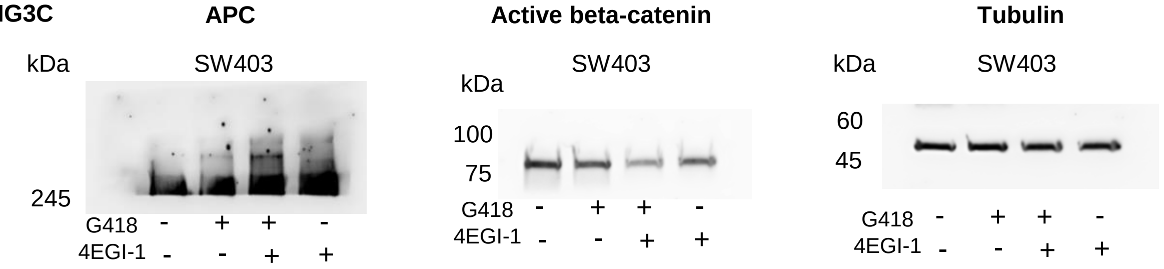

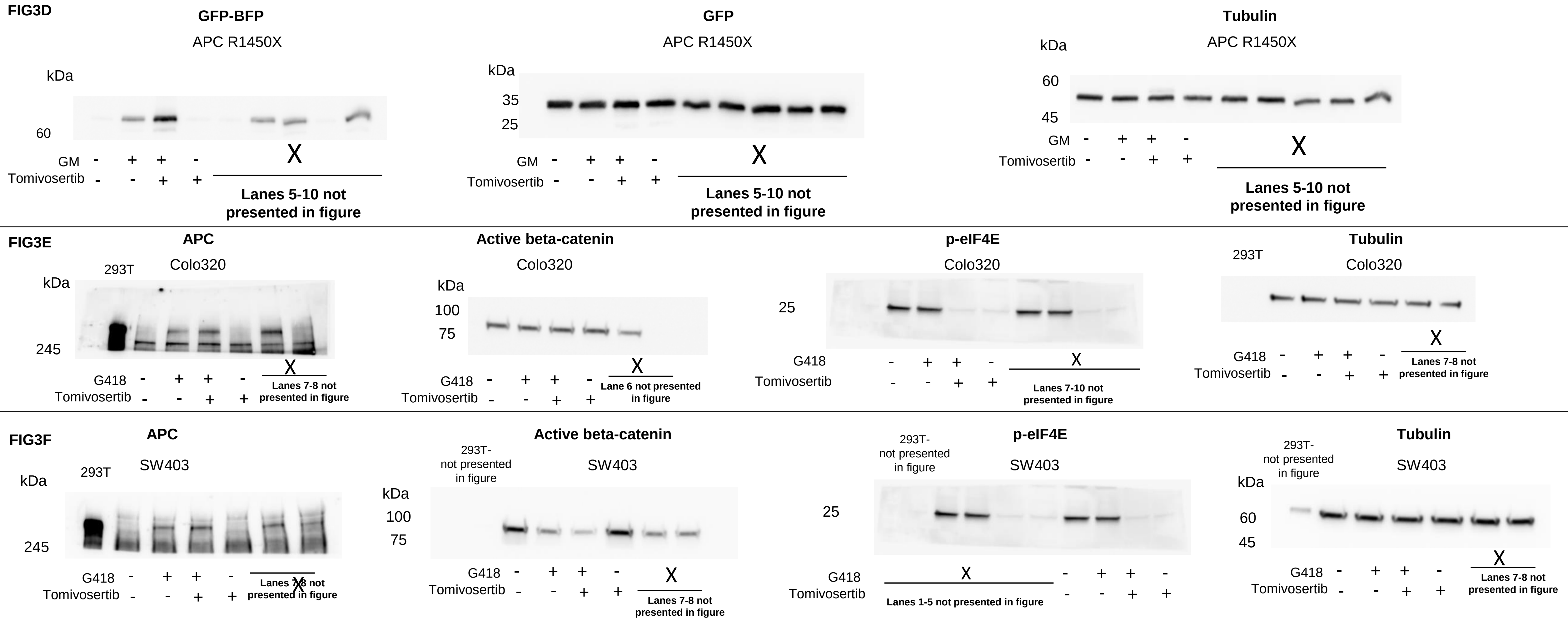
FIG3D
GFP-BFP
APC R1450X
kDa
60
GM - + + -
Tomivosertib - - + +
X
Lanes 5-10 not presented in figure
GFP
APC R1450X
kDa
35
25
GM - + + -
Tomivosertib - - + +
X
Lanes 5-10 not presented in figure
Tubulin
APC R1450X
kDa
60
45
GM - + + -
Tomivosertib - - + +
X
Lanes 5-10 not presented in figure
FIG3E
APC
293T
Colo320
kDa
245
G418 - + + -
Tomivosertib - - + +
X
Lanes 7-8 not presented in figure
Active beta-catenin
Colo320
kDa
100
75
G418 - + + -
Tomivosertib - - + +
X
Lane 6 not presented in figure
p-eIF4E
Colo320
25
G418 - + + -
Tomivosertib - - + +
X
Lanes 7-10 not presented in figure
Tubulin
293T
Colo320
G418 - + + -
Tomivosertib - - + +
X
Lanes 7-8 not presented in figure
FIG3F
APC
293T
SW403
kDa
245
G418 - + + -
Tomivosertib - - + +
X
Lanes 7-8 not presented in figure
Active beta-catenin
293T- not presented in figure
SW403
kDa
100
75
G418 - + + -
Tomivosertib - - + +
X
Lanes 7-8 not presented in figure
p-eIF4E
293T- not presented in figure
SW403
25
G418
Tomivosertib
X
Lanes 1-5 not presented in figure
- + + -
- - + +
Tubulin
293T- not presented in figure
SW403
kDa
60
45
G418 - + + -
Tomivosertib - - + +
X
Lanes 7-8 not presented in figure

FIG4A

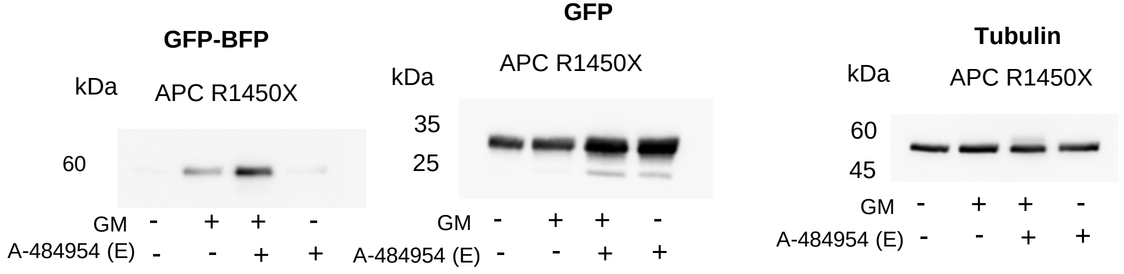

FIG4B

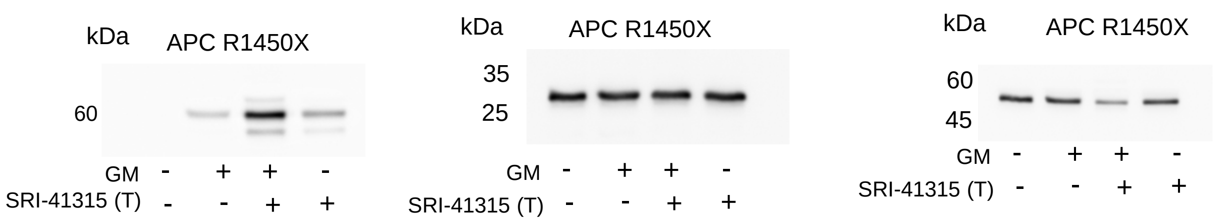

FIG4C

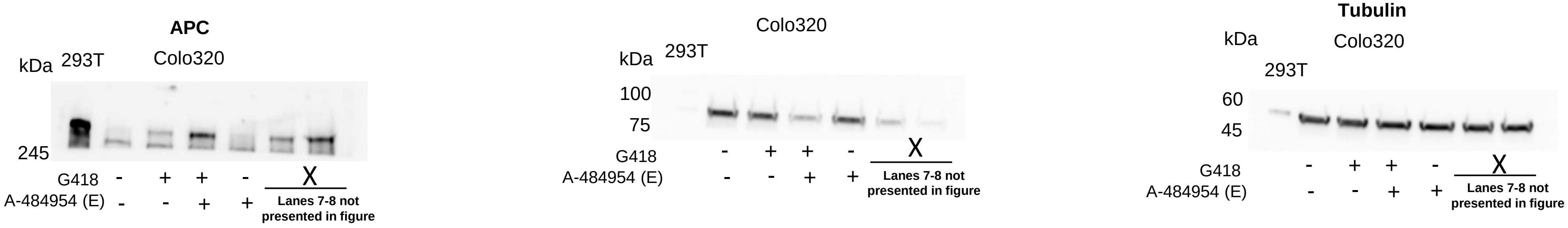

FIG4D

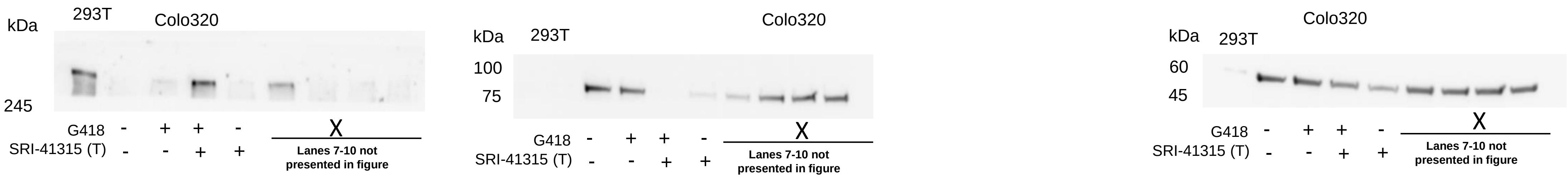

FIG5A

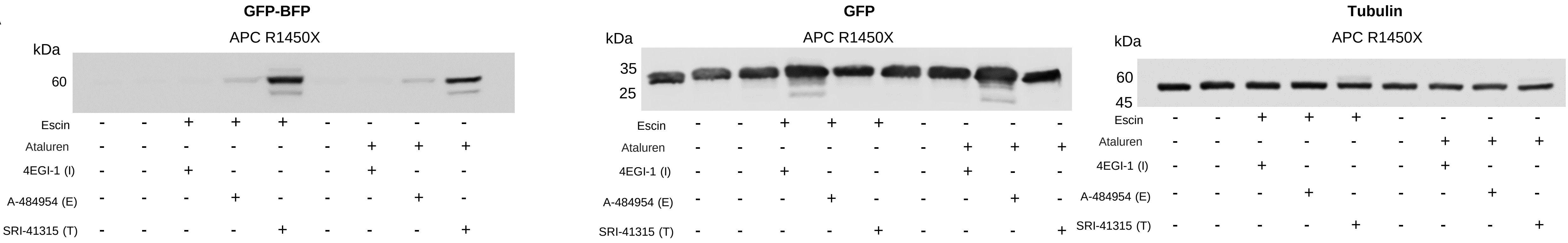

GFP-BFP
APC R1450X
kDa
60
GFP
APC R1450X
kDa
35
25
Tubulin
APC R1450X
kDa
60
45
Escin
Ataluren
4EGI-1 (I)
A-484954 (E)
SRI-41315 (T)

FIG5B

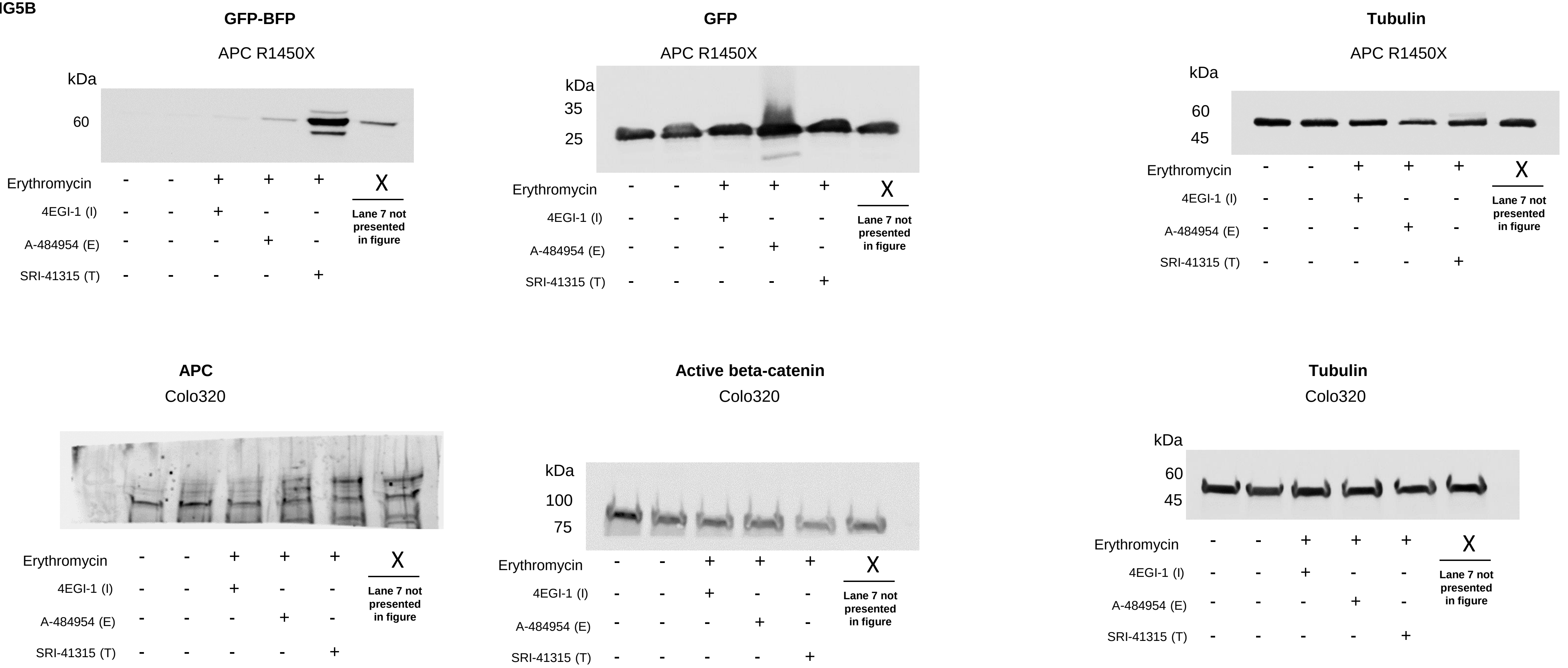

GFP-BFP
APC R1450X
kDa
60
GFP
APC R1450X
kDa
35
25
Tubulin
APC R1450X
kDa
60
45
APC
Colo320
Active beta-catenin
Colo320
kDa
100
75
Tubulin
Colo320
kDa
60
45
Erythromycin
4EGI-1 (I)
A-484954 (E)
SRI-41315 (T)
X
Lane 7 not presented in figure

FIGS1A

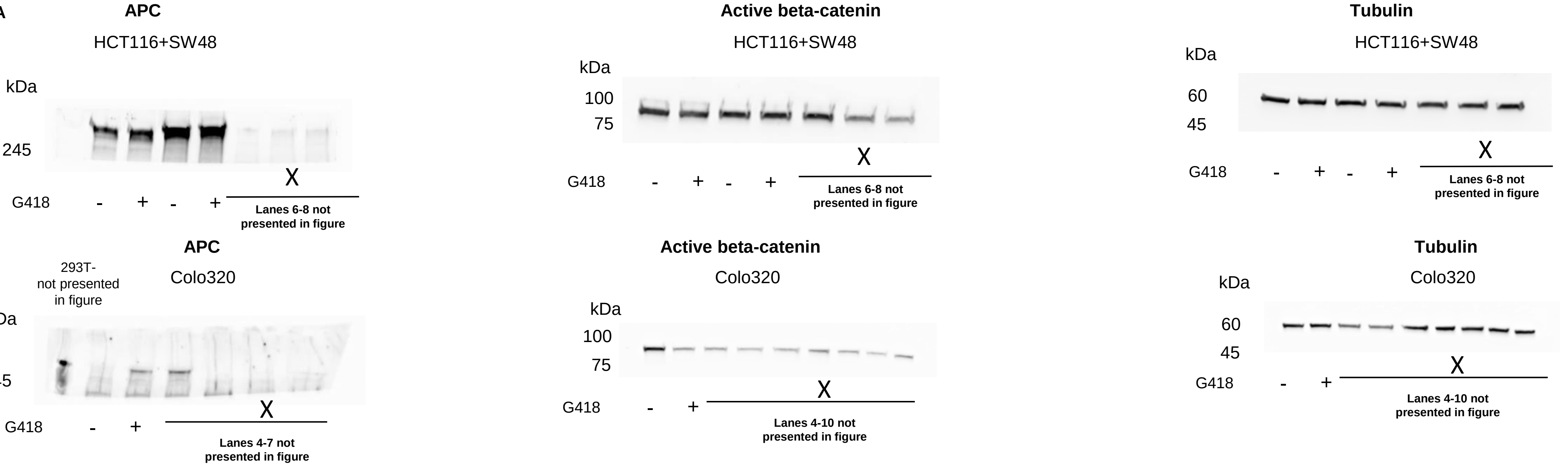

FIGS1B

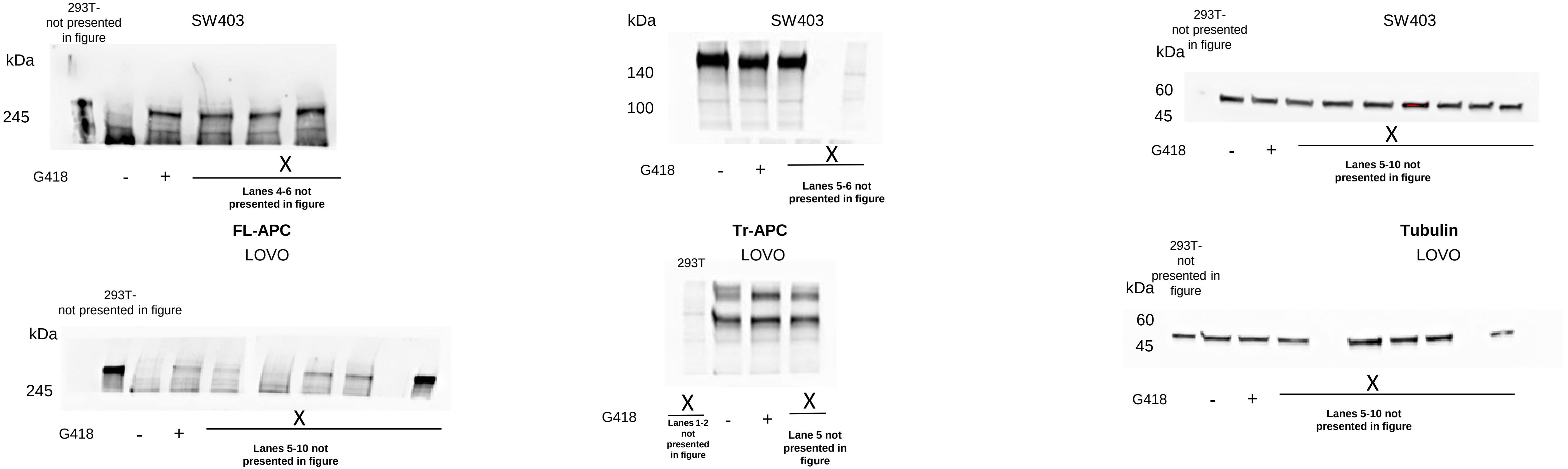

FIGS1C

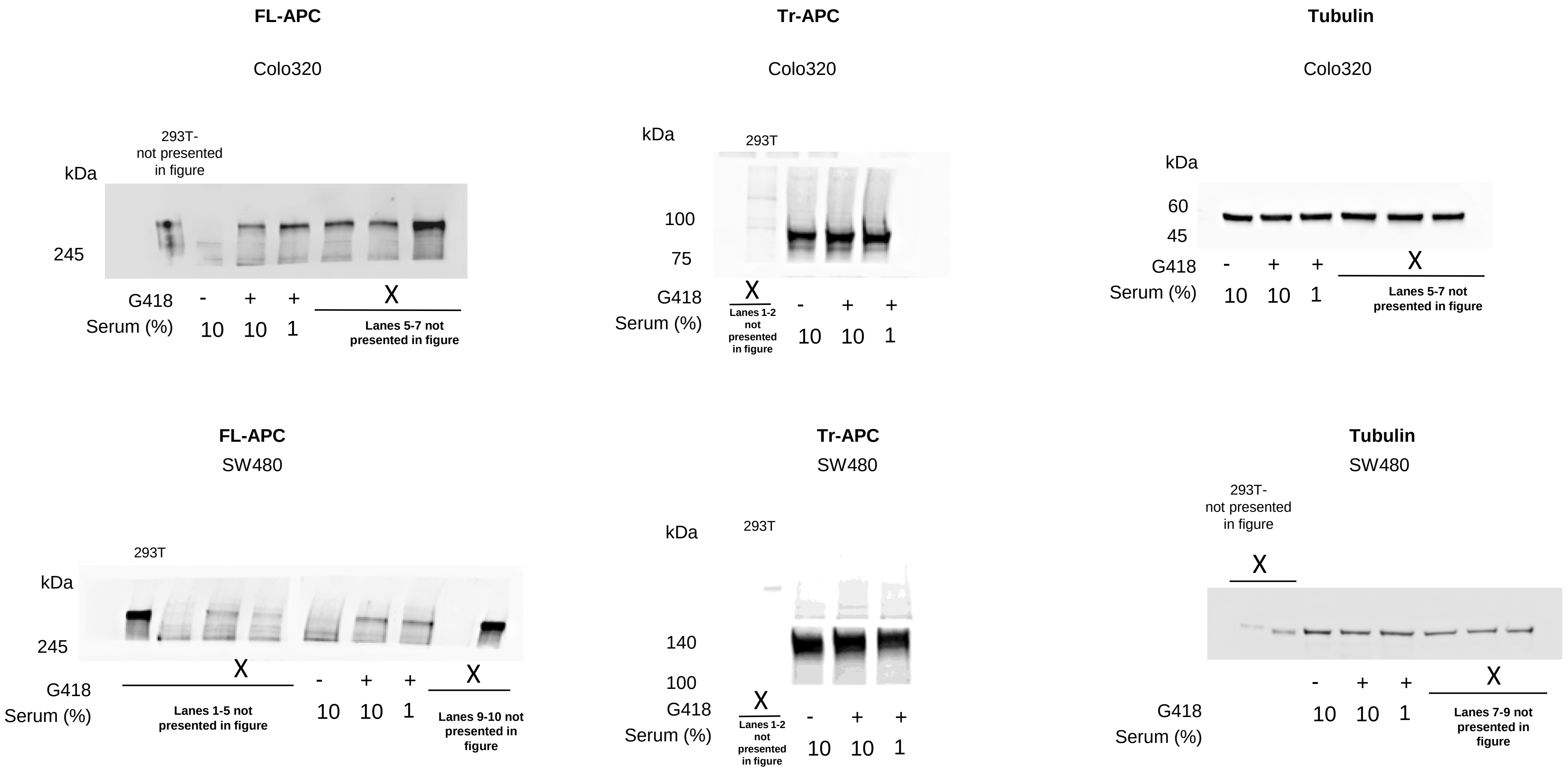
FL-APC
Colo320
293T-
not presented
in figure
kDa
245
G418
-
+
+
X
Serum (%)
10
10
1
Lanes 5-7 not presented in figure
Tr-APC
Colo320
kDa
293T
100
75
G418
X
Lanes 1-2 not presented in figure
-
+
+
Serum (%)
10
10
1
Tubulin
Colo320
kDa
60
45
G418
-
+
+
X
Serum (%)
10
10
1
Lanes 5-7 not presented in figure
FL-APC
SW480
293T
kDa
245
X
G418
-
+
+
X
Serum (%)
Lanes 1-5 not presented in figure
10
10
1
Lanes 9-10 not presented in figure
Tr-APC
SW480
kDa
293T
140
100
X
G418
Lanes 1-2 not presented in figure
-
+
+
Serum (%)
10
10
1
Tubulin
SW480
293T-
not presented
in figure
X
-
+
+
X
G418
Serum (%)
10
10
1
Lanes 7-9 not presented in figure

FIGS2A

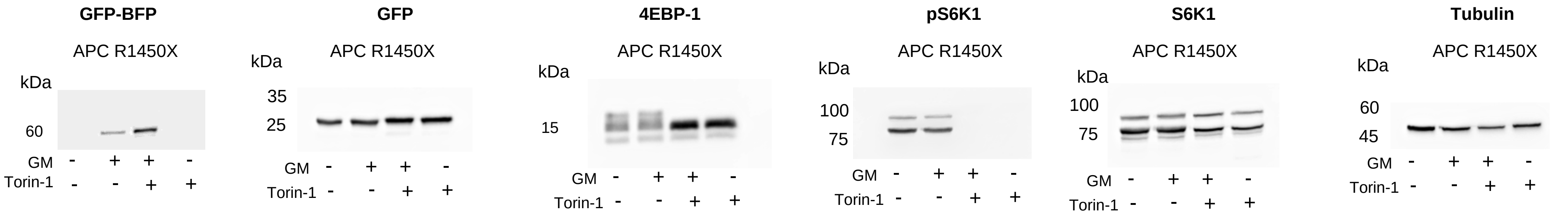

FIGS2B

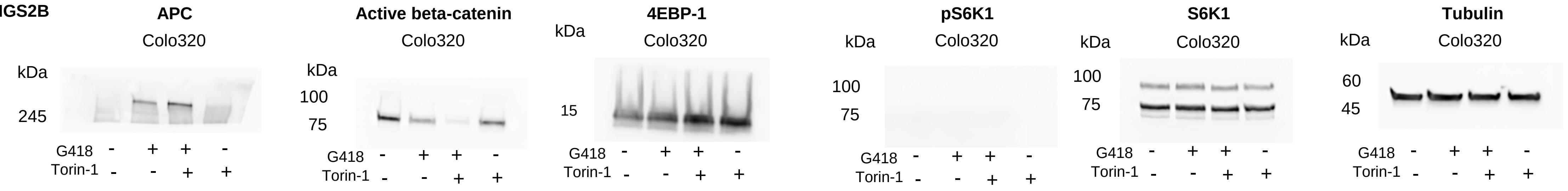

FIGS2C

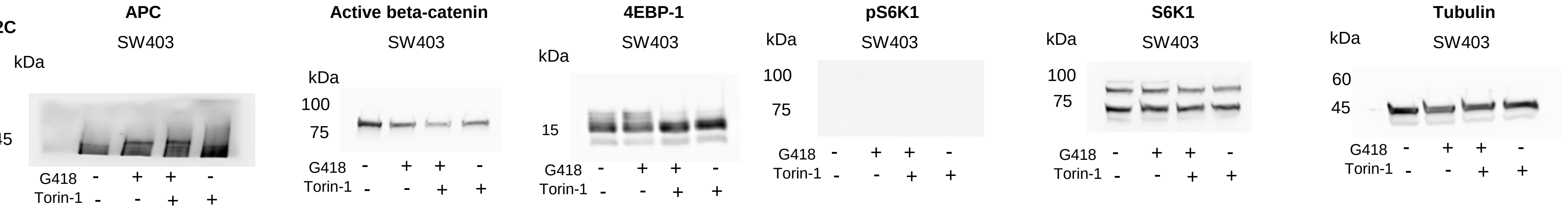

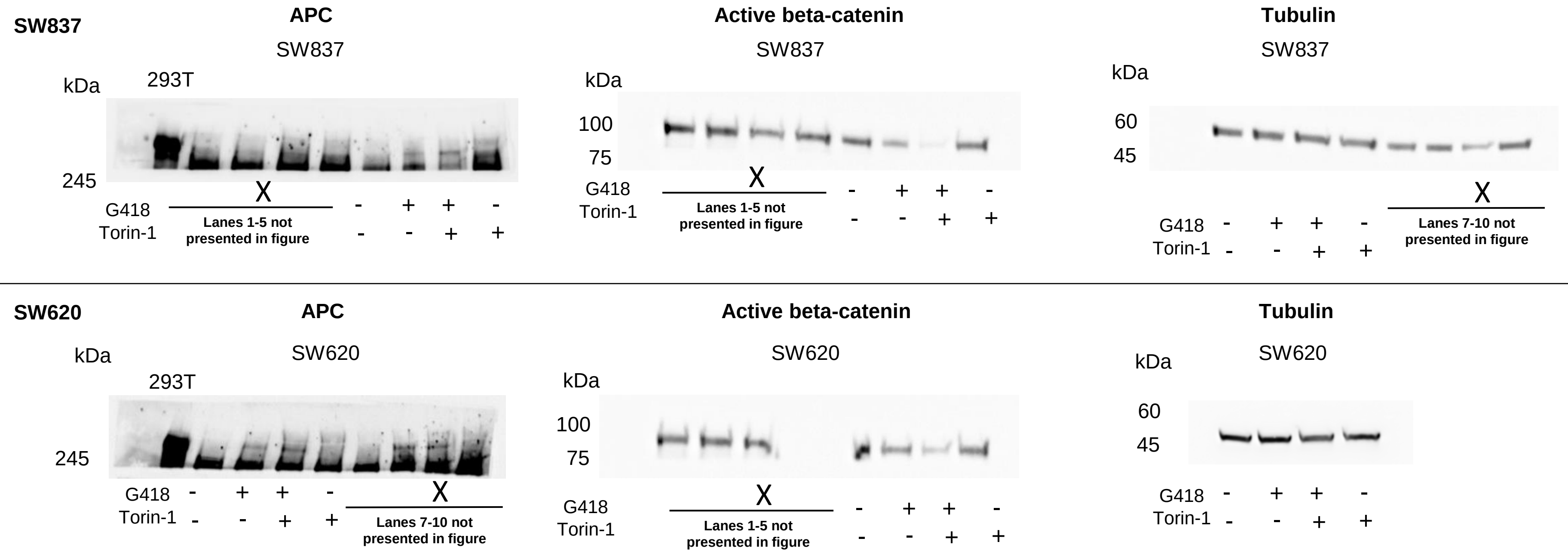
SW837
APC
SW837
kDa
293T
245
X
G418
Torin-1
Lanes 1-5 not presented in figure
- + + -
- - + +
Active beta-catenin
SW837
kDa
100
75
G418
Torin-1
X
Lanes 1-5 not presented in figure
- + + -
- - + +
Tubulin
SW837
kDa
60
45
G418
Torin-1
- + + -
- - + +
X
Lanes 7-10 not presented in figure
SW620
APC
kDa
SW620
293T
245
G418
Torin-1
- + + -
- - + +
X
Lanes 7-10 not presented in figure
Active beta-catenin
SW620
kDa
100
75
G418
Torin-1
X
Lanes 1-5 not presented in figure
- + + -
- - + +
Tubulin
SW620
kDa
60
45
G418
Torin-1
- + + -
- - + +

FIGS7

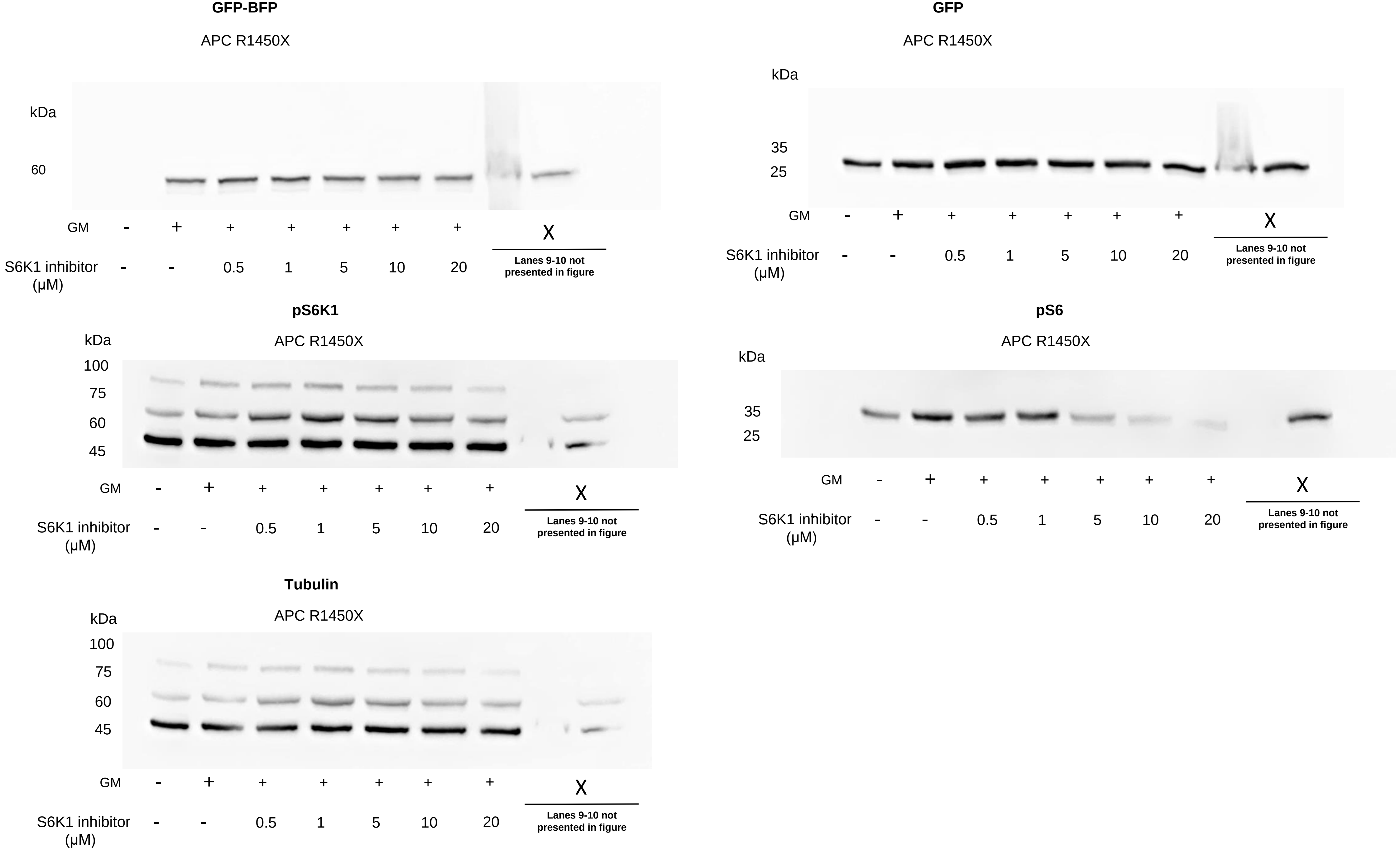

GFP-BFP
APC R1450X
kDa
60
GM - + + + + + +
S6K1 inhibitor (µM) - - 0.5 1 5 10 20
X
Lanes 9-10 not presented in figure
GFP
APC R1450X
kDa
35
25
GM - + + + + + +
S6K1 inhibitor (µM) - - 0.5 1 5 10 20
X
Lanes 9-10 not presented in figure
pS6K1
kDa
APC R1450X
100
75
60
45
GM - + + + + + +
S6K1 inhibitor (µM) - - 0.5 1 5 10 20
X
Lanes 9-10 not presented in figure
pS6
APC R1450X
kDa
35
25
GM - + + + + + +
S6K1 inhibitor (µM) - - 0.5 1 5 10 20
X
Lanes 9-10 not presented in figure
Tubulin
kDa
APC R1450X
100
75
60
45
GM - + + + + + +
S6K1 inhibitor (µM) - - 0.5 1 5 10 20
X
Lanes 9-10 not presented in figure

FIGS8A-Apidaecin

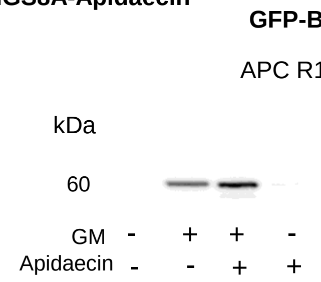

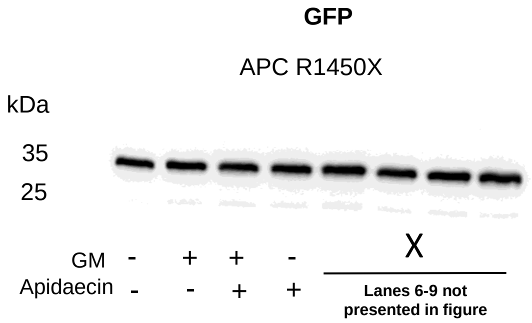

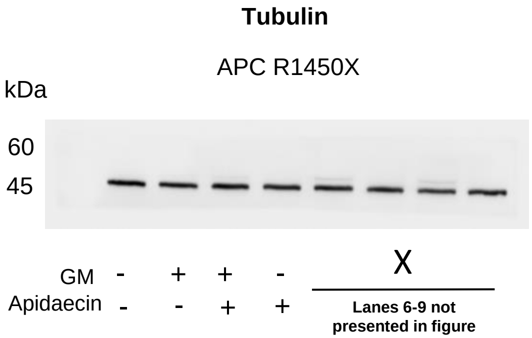

FIGS8A-NOG

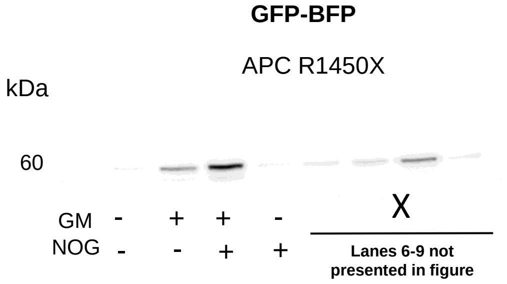

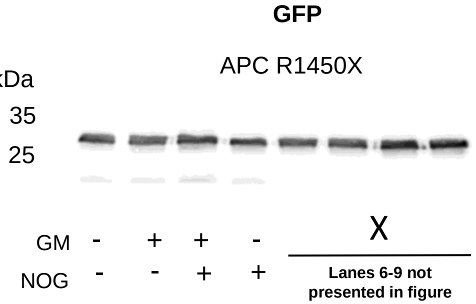

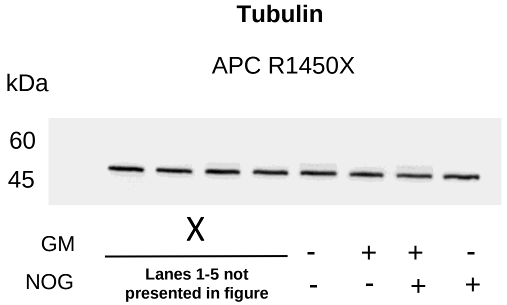

Supplement: S1 Raw Images — (PDF) [file pbio.3002355.s010.pdf]
