# Supplementary material for: Nonsense mutation suppression is enhanced by targeting different stages of the protein synthesis process
Source: PLoS Biol. 2023 Nov 9;21(11):e3002355. doi: 10.1371/journal.pbio.3002355 (PMC10684085; doi:10.1371/journal.pbio.3002355)
Supplement: Attachment — Submitted filename: Rebuttle .pdf [file pbio.3002355.s011.pdf]

We would like to thank the Reviewers for their insightful comments that have helped us improve our manuscript. Please find below our response to the specific comments. In the revised manuscript, all major changes are marked in green.
Importantly, we focused on understanding the mechanisms underlining the effects that lead to enhanced readthrough induced by the antibiotics and provided data showing that modulating different stages of the protein translation machinery play a key role in this effect.

Reviewer #1

We thank the Reviewer for stating that "Overall the article is well structured, the experiments are clearly and logically described. The data is convincing and there does not seem to be a sticking point", and also for pointing to the fact that "This manuscript will therefore be of interest to people working in the field of readthrough, therapeutic approaches and genetic diseases, but also more generally to people studying gene translation and expression".

Specific comments:

In chapter "Serum starvation increased APC nonsense-mutation readthrough in CRC cell lines", last line please replace "sratvation" by "starvation".

The mistake has been corrected.

In chapter "Torin-1 increases antibiotic-mediated nonsense codon readthrough", 6 lines before the end please replace "Totin-1" by "Torin-1"

The mistake has now been corrected.

The following sentence in the discussion has to be rewritten because NMD degrades RNA and not proteins: "In many cases, the cancer cells express a truncated APC protein that is not degraded by the NMD as most of the nonsense mutations occur in a hotspot within the last APC exon, thus they are not recognized by the exon junction complex method of NMD [55] ".

The sentence has been corrected and rephrased to say: "Mutated APC transcripts are often NMD-resistant as most of the nonsense mutations occur in a hotspot within the last APC exon and therefore not recognized by the exon junction complex that induces NMD".

Change "combitation" into "combination" 7 lines from the end of the discussion.

The mistake has been corrected.

Figure 5, the authors analyze the effect of an inhibition of the activity of eIF4E using the small molecule 4EGI-1. They are testing for an endogenous nonsense mutation in the APC gene in COLO320 cells. To be consistent with Figure 4, the authors should also show the same effect on SW403 cells.

*The requested missing experiment has been added **[Fig 3C and S6B]** and the results are discussed.*

Reviewer #2

We thank the Reviewer for acknowledging the “nice flow of the paper” and that “The involvement of mTOR pathway in PTC RT is interesting”. We have addressed the Reviewer’s comments and added the requested experiments as follows:

Major comments -
1- My major concern is about the concentration of G418 that authors used in their PTC RT experiments. G418 at 1.5 mg/ml is extremely high and usually very toxic in many cell types. We have observed that in the presence of high levels of aminoglycosides, PTC RT enhances significantly at the cost of severe toxicity. Authors should be careful to avoid such toxicity. Showing the viability (live cells as well as apoptosis levels, both) in cells (stable and CRC cells) at 24 and also 48 hours post-treatment (G418, Torin-1, Rapamycin and their combination) must be performed as an indicator of cellular health.

*We thank the Reviewer for raising this point. In the revised manuscript, most experiments were conducted with a lower dose of antibiotics (500µg/ml). We have used the G418 at 1.5 mg/ml only when comparing it to our previous results showing the effects of serum starvation on readthrough, where this high concentration was used [1] **(Fig. 1)** and when using immunofluorescent experiments on colo320 and SW403 cells **(Fig. 2E)**. In all other experiments 500µg/ml G418 was used. We have now tested cell viability under the different treatments, using the 500ug/ml dose **(Fig. S3),** demonstrating that cell survival is between 60%-100% under different conditions.*

2- Control cell lines (a CRC cell line without APC mutation to show WT levels of APC, and a CRC cell line with APC mutations other than PTC as negative control) must be included to the experiments. It is much better to report the level of PTC readthrough relative to WT rather than untreated mutant cells. Regarding the low level of PTC RT enhancement in combination treatment it is good to know whether these levels have any biological significance when compared to normal APC levels.

*We have now added the requested missing control cells to the manuscript **(Fig. S1A)**: <u>HCT116</u>, which harbors a β-catenin mutation (and wild-type APC), and <u>SW48</u> expressing an APC gene with a missense mutation. APC is mostly unaffected by the enhancing readthrough treatment in these cell lines. Please note that the endogenous expression levels of APC in these cells are higher than those achieved by restoring APC levels in Colo320 cells. Although the induced APC restoration is relatively minor, the effect on reducing active β-catenin levels is significant. The levels of induced readthrough depend on different factors such as the type of the stop codon, the surrounding sequence and the gene itself [2, 3]. As the Reviewer stated, it is important to determine what is the minimal level of full-length protein induced by the readthrough treatment that has therapeutic effects. It has been shown that this level is different in each protein and disease. For example, even 1 % of normal*

protein function in lysosomal storage disease may restore a near-normal or clinically less severe phenotype [4, 5]. For cystic fibrosis, 10 to 35 % of CFTR activity might be needed to alleviate pulmonary morbidity Field significant [7], and in DMD – 1-30% of the full-length dystrophin is needed [6]. Similarly, our results indicate that even if we can restore only relatively low amounts of the APC protein [1], these levels may *have beneficial therapeutic effects [8]. This important point has now been added to the introduction of the revised manuscript.*

3- In the introduction section the authors mentioned that "there is increasing evidence that APC truncations may exert dominant functions contributing to colon tumorigenesis. These include enhancement of cell migration, interference with spindle formation, and induction of chromosome instability [35-38]." Usually in the course of PTC readthrough the truncated protein is also increased (Baradaran-Heravi et al, 2016, Nucleic Acids Research). In this study, in addition to full-length APC, authors need to show the truncated form in the CRC cell lines and find out whether this form also increases during mTOR inhibition and G418 treatment. Since the dominant function of APC truncation contributes to colon tumorigenesis, would an increase in truncated protein during PTC readthrough be considered an adverse side effect?

We have now conducted the missing experiment. In revised Figs. ***(Fig. S1B-C)*** *we show that the increase in full-length APC following nonsense mutation-induced readthrough is not observed in the truncated APC protein product. Truncated APC is known to be NMD-resistant [9] and thus accumulates in cancers that originate from APC-premature termination codons. p53, on the other hand, is highly affected by NMD (as discussed in Baradaran-Heravi et al, 2016, Nucleic Acids Research), and thus nonsense mutation readthrough, which leads to prolonged ribosomal protection of the p53 transcripts, could affect the low levels of the truncated p53 protein product.*

4- I am wondering how the authors reconcile diminished translation initiation and increased PTC readthrough? What is the author's proposed model?

We agree that this is a very important point. Our results show that 4EGI-1 that affects translation initiation, enhances-nonsense mutation readthrough only in the presence of aminoglycosides ***(Fig. 3)****. Moreover, we have now added another invitation inhibitor- an MNK inhibition that impedes eIF4E phosphorylation (****Fig. 3D-F****) that shows similar effects to those of 4EGI-1 on enhancement of antibiotic mediated readthrough. Aminoglycosides exert their PTC readthrough activity by binding at the eukaryotic ribosome's decoding center and reducing translation termination factors' ability to accurately recognize the PTC Field [10, 11]. Similarly to our results, It has been shown that other compounds, such as the small molecules CDX5-1 [12] or the drug mefloquine [13] that do not show readthrough activity when used as single agents potentiate the readthrough activity of aminoglycoside possibly by directly targeting the translation machinery although the exact mechanism is still unclear and should be further studied. We speculate that changes in the dynamics of the ribosomes bound to the mRNA that are affected by these inhibitors may lead to enhanced stalling and misreading induced by the antibiotic. Our preliminary (not shown) results suggest that ribosomal collision does not affect this process. We are currently conducting additional*

experiments to understand the exact mechanism by which initiation-inhibition enhances the activity of aminoglycosides on nonsense mutation readthrough.

5- In figure 2C, can authors induce Gentamicin related PTC RT in TSC-/- cells by treating them with Torin-1 or Rapamycin or 4EGI-1? Please show the results.

The requested missing data has been added to the Figure ***(Fig. 2A)*** *and corresponding text.*

6- Please show the APC mRNA levels in CRC cell lines and discuss its changes in different treatment combinations.

*We have now measured the APC mRNA levels under the different treated combinations and have added the results to the revised manuscript (**Fig. S5**). These results have been discussed (in the result mTOR inhibition increases antibiotic-induced nonsense mutation readthrough)*
) as follows: "Interestingly, although mutated APC transcripts are relatively stable, a slight increase in mRNA levels was observed in treated Colo320 cells as opposed to SW403 where mRNA transcripts were unaffected by readthrough or readthrough enhancement".

7- It would be nice to see the effect of combination treatment on PTC RT response in other CRC cell lines they discussed in Figure 2A.

We have now added two CRC cell lines, chosen from the group of cells where serum starvation enhances readthrough. These cells respond to the combination treatment on PTC readthrough (SW837 and SW620; ***Fig. S4)****.*

Minor comments-

1- It would be nice to explain in more detail the GFP-BFP cell line when the authors mention it for the first time.

A detailed explanation on the GFP-BFP reporter plasmin has been added to the revised manuscript (in the results section, under the paragraph - The mTOR pathway may regulate antibiotic-induced nonsense mutation readthrough (mTOR inhibition increases antibiotic-induced nonsense mutation readthrough).

2- In figure 2A, how many proteins did they end up analyzing? Please mention the number.–

We tested 214 proteins where we had the data for all 8 cell lines examined. Out of these proteins, 8 were statistically significantly different. 4EPB-1 and its three phosphorylated forms had the most statistical significance out of these proteins. This information has now been added to the text.

3- Authors mentioned, "As can be seen, Totin-1 induced APC restoration in both cell lines, though the re-expression of full-length APC was more complete in COLO320 cells". What do they mean by "complete" when they do not have WT levels of APC to compare with? Do

they mean "more efficient" ?

We apologize for the confusing terminology. We compared the readthrough activity to the null condition and not the wild-type expression. The sentence has been completely rephrased in the discussion paragraph.

4- Please provide the full image of APC western blots to better visualize to full length and truncated forms in one blot.

*Figures **S1B-C** now show both full-length-APC and truncated APC in untreated and treated cells. Technically, due to the differences in protein sizes (90-160kDa for the truncated APC protein product in the different cell lines and full-length APC-312kDa) and the poor quality of the available antibodies, both APC forms cannot be detected on the same blot and were thus analyzed on separate gels.*

5- In figure 5, please add 4EGI-1 treatment (alone) lane for both panels. Also, please add quantification of active β-catenin for panel B.

*The experiments have been repeated and this missing data has been added to the figure **(Fig. 3A-C, and S6)** and corresponding text.*

6- In the discussion, it is said "As all the CRC cells that responded to mTOR inhibition by increased PTC readthrough show high levels of 4E-PB1 we conclude that inhibiting cap-dependent protein translation initiation enhances antibiotic-mediated PTC readthrough". This statement is not accurate. The authors have tested only one cell line, COLO320, which has high 4E-PB1 expression and responds to mTOR inhibition in terms of increased PTC RT.

*This statement has been changed and corrected to state that: "4EBP-1 and its three phosphorylated forms were highly expressed in the cells that demonstrated increased antibiotic induced-readthrough of nonsense mutations following serum depletion **(Fig. 1C)**".*

<u>**Referees cross-commenting**</u>
I appreciate reviewer #1 and #3 comments and I also agree about the nice flow of the paper. We routinely study G418 effect on PTC readthrough in many different cell lines. My major reservation is about the concentration of G418 that authors used in their PTC RT experiments. G418 at 1.5 mg/ml is extremely high and usually very toxic in many cell types. We have observed that in the presence of high levels of aminoglycosides, PTC RT enhances significantly at the cost of severe toxicity. Authors should be careful to avoid such toxicity. Showing the viability (live cells as well as apoptosis levels, both) in cells (stable and CRC cells) at 24 and also 48 hours post treatment (G418, Torin-1, Rapamycin and their combination) must be performed as an indicator of cellular health.

Thank you for this comment. As described above in response to Reviewer #2 comments, the majority of our experiments were now conducted with a lower dose of antibiotics (500ug/ml). Although Reviewer #3 mentioned that “some cell types can tolerate those

doses", we have now tested the survival of the various treatments, using the 500ug/ml dose ***(Fig. S3)*** *and demonstrated that cell death did not exceed 40% under any condition. Only viable cells were used in our experiments.*

The involvement of mTOR pathway in PTC RT is interesting; however, I am not sure about the biological value of this finding as mTOR inhibition marginally enhances aminoglycoside induced PTC RT (2-2.5-fold in COLO320 cells). Also, the number of cell lines tested in this manuscript is *limited to only* two CRC cell lines which makes the interpretation of the results more difficult.

To address this important point additional CRC cell lines have now been used throughout the manuscript. As different studies show that increasing nonsense mutation readthrough levels and inducing some restoration of the full-length protein, even by small amounts could have beneficial value (please see our response to Reviewer # 2, point 2) we suggest that enhancing nonsense mutation readthrough by inhibiting the mTOR pathway may have therapeutic value. We have now emphasized in the manuscript that the different strategies for inducing readthrough (including ours) do not achieve wild-type levels and that this point needs to be considered when evaluating the therapeutic potential of this treatment strategy.

Reviewer #3

We thank the Reviewer for stating that "This is an important finding". We have addressed the specific Reviewer's comments as follows:

1) In the first paragraph of the Results Section, you use serum starvation to enhance readthrough. However, I could not find how long you maintained serum starvation, whether it was added before or concurrently to aminoglycoside addition, etc. Please clarify this point.

We apologize for omitting this point. The treatment conditions of serum starvation have now been added to the results section and to the legend (cells were incubated for 24 h in a medium containing 10% or 1% serum supplemented with 1.5mg/ml G418).

2) Fig. S1: I can't read the x-axis labels. Please fix this.

The figure has been corrected (currently ***Fig. 1)****.*

3) First paragraph in the torin-1 section: you don't refer the reader to Fig 3B and 3C. I suggest that you revise the text as follows: "Next, the effect of mTOR inhibition on antibiotic-mediated endogenous APC readthrough in the CRC cell lines COLO320 (Fig. 3B) and SW403 (Fig. 3C) was examined where aminoglycosides induced relatively high levels of APC restoration".

The text has been revised and corrected.

4) In figs. 3, 4 and 5, you label the panels using the cell lines COLO320 (panel B) and SW403 (panel C), but not for the APC R1450X line (panel A). The reason for this omission is not

clear, but it would help the reader follow your work if you added it.

The missing panels have now been labeled correctly.

5) You don't mention Fig. S3 in the text of the manuscript. Please add a sentence to the last paragraph of the Results since it is important to note that 4EGI-1 does not induce readthrough alone.

The Figure has now been mentioned and the finding that 4EGI-1 does not induce readthrough alone is now shown in ***(Fig. 3)*** *(please see our response to Reviewer #2, point 5, minor points section).*

<u>**Referees cross-commenting**</u>
I agree that 1.5 mM G418 sounds high, but some cell types can tolerate those doses. Controls to examine toxicity seem appropriate and won't take too long. In addition, one panel showing that the mTOR inhibition also stimulate readthrough at a lower G418 dose would help to allay this concern.

Please see our response to this point above (Reviewer #2, point 1). In the current manuscript, all experiments except ***(Fig. 1A & Fig. 2E)*** *were conducted with 500ug/ml G418.*

<u>References</u>

[1] A. Wittenstein, M. Caspi, Y. David, Y. Shorer, P.T. Nadar-Ponniah, R. Rosin-Arbesfeld, Serum starvation enhances nonsense mutation readthrough, J Mol Med (Berl), 97 (2019) 1695-1710.
[2] C. Floquet, I. Hatin, J.P. Rousset, L. Bidou, Statistical analysis of readthrough levels for nonsense mutations in mammalian cells reveals a major determinant of response to gentamicin, PLoS Genet, 8 (2012) e1002608.
[3] L. Martorell, V. Cortina, R. Parra, J. Barquinero, F. Vidal, Variable readthrough responsiveness of nonsense mutations in hemophilia A, Haematologica, 105 (2020) 508-518.
[4] I. Maire, Is genotype determination useful in predicting the clinical phenotype in lysosomal storage diseases?, J Inherit Metab Dis, 24 Suppl 2 (2001) 57-61; discussion 45-56.
[5] I. Nudelman, D. Glikin, B. Smolkin, M. Hainrichson, V. Belakhov, T. Baasov, Repairing faulty genes by aminoglycosides: development of new derivatives of geneticin (G418) with enhanced suppression of diseases-causing nonsense mutations, Bioorg Med Chem, 18 (2010) 3735-3746.
[6] M. Dabrowski, Z. Bukowy-Bieryllo, E. Zietkiewicz, Advances in therapeutic use of a drug-stimulated translational readthrough of premature termination codons, Mol Med, 24 (2018) 25.
[7] E. Kerem, Pharmacologic therapy for stop mutations: how much CFTR activity is enough?, Curr Opin Pulm Med, 10 (2004) 547-552.

[8] R. Kariv, M. Caspi, N. Fliss-Isakov, Y. Shorer, Y. Shor, G. Rosner, E. Brazowski, G. Beer, S. Cohen, R. Rosin-Arbesfeld, Resorting the function of the colorectal cancer gatekeeper adenomatous polyposis coli, Int J Cancer, 146 (2020) 1064-1074.
[9] R.G. Lindeboom, F. Supek, B. Lehner, The rules and impact of nonsense-mediated mRNA decay in human cancers, Nat Genet, 48 (2016) 1112-1118.
[10] B. Francois, R.J. Russell, J.B. Murray, F. Aboul-ela, B. Masquida, Q. Vicens, E. Westhof, Crystal structures of complexes between aminoglycosides and decoding A site oligonucleotides: role of the number of rings and positive charges in the specific binding leading to miscoding, Nucleic Acids Res, 33 (2005) 5677-5690.
[11] N. Garreau de Loubresse, I. Prokhorova, W. Holtkamp, M.V. Rodnina, G. Yusupova, M. Yusupov, Structural basis for the inhibition of the eukaryotic ribosome, Nature, 513 (2014) 517-522.
[12] A. Baradaran-Heravi, A.D. Balgi, C. Zimmerman, K. Choi, F.S. Shidmoossavee, J.S. Tan, C. Bergeaud, A. Krause, S. Flibotte, Y. Shimizu, H.J. Anderson, V. Mouly, E. Jan, T. Pfeifer, J.B. Jaquith, M. Roberge, Novel small molecules potentiate premature termination codon readthrough by aminoglycosides, Nucleic Acids Res, 44 (2016) 6583-6598.
[13] M.W. Ferguson, C.A.N. Gerak, C.C.T. Chow, E.J. Rastelli, K.E. Elmore, F. Stahl, S. Hosseini-Farahabadi, A. Baradaran-Heravi, D.M. Coltart, M. Roberge, The antimalarial drug mefloquine enhances TP53 premature termination codon readthrough by aminoglycoside G418, PLoS One, 14 (2019) e0216423.
[14] H. Wang, F. Huang, J. Wang, P. Wang, W. Lv, L. Hong, S. Li, J. Zhou, The synergistic inhibition of breast cancer proliferation by combined treatment with 4EGI-1 and MK2206, Cell Cycle, 14 (2015) 232-242.
