# Supplementary material for: Nonsense mutation suppression is enhanced by targeting different stages of the protein synthesis process
Source: PLoS Biol. 2023 Nov 9;21(11):e3002355. doi: 10.1371/journal.pbio.3002355 (PMC10684085; doi:10.1371/journal.pbio.3002355)
Supplement: Attachment — Submitted filename: Response to Reviewers.docx [file pbio.3002355.s013.docx]

We would like to thank all the Reviewers for their insightful comments that have helped us improve our manuscript and broaden the scope of the paper. Please find below our response to the specific comments. In the revised manuscript, all changes are marked in yellow.

Importantly, we extended our experiments to include non-aminoglycosides readthrough-inducers to generalize the impact of the different translation steps on nonsense suppression. As suggested by the Editor and the Reviewer, these results generalize the findings and strengthen the overall message of the manuscript and thus were added as an additional figure to the main text.

We would like to thank Reviewer # 3 for stating that “*The authors were very responsive to the reviewers' comments and have addressed all my concerns about this manuscript*”.

Reviewer #1:

We thank the Reviewer for stating, "*Overall, the article is clear, the experiences are well described and organized in a coherent way. This article provides very interesting and useful data for the scientific community”.*

The important comments raised by the Reviever have been addressed as follows:

1*) Figure 1A, the authors indicate that to test the functionality of APC restored by readthrough, they measure the expression level of beta-catenin. If we can clearly see that as soon as APC is increased, the level of beta-catenin decreases, it is however not proportional to the level of expression of APC. For example, in SW837 and SW620 cells at 1% serum, the level of beta-catenin is not lower than the 10% serum condition although the amount of APC produced is higher. The authors should comment on this point which is perhaps relative to a threshold of beta-catenin below which one cannot go?*

Thank you for this comment. This is an important point, and different publications have shown that the levels of β-catenin do not directly correlate to the levels of full-length APC (for example, as shown in [1-5]), and as the Reviewer has noted, this phenomenon is indeed thought to represent a threshold of β-catenin expression. Moreover, it was shown that the number of β-catenin binding sites in the APC protein affects its ability to decrease β-catenin levels, though not in an absolute correlation [6]. Our results using the Colo320 cells in which, uniquely, the APC has no β-catenin binding sites [7, 8] show the strongest effect on β-catenin expression levels [9]. SW837 and SW620 still retain a few β-catenin binding sites (SW620: three β-catenin binding sites and one β-catenin binding and downregulation site; SW837: three β-catenin binding sites and two β-catenin binding and downregulation sites [8, 10, 11]), and thus, restored full-length APC may not directly affect the expression of β-catenin. For this reason, in most experiments (Figs. 2-5 in the revised manuscript), the Colo320 cells were used.

In addition, other mechanisms, such as neddylation [12], GSK3β mTOR-dependent phosphorylation [13], and microRNAs' expression [14], that have been shown to be involved in regulating β-catenin levels may be influenced by the readthrough-inducing drugs and restrict the effects on β-catenin.

This important point has now been addressed in the revised manuscript (page 7, upper part).

*2)  Figure S3: the curve representation of the effect of different treatments on cell survival seems to me inappropriate because there is no continuum between the treatments. A representation in histogram would seem more appropriate.*

The graph and legend have been corrected (Revised Fig. S3).

*3) Figure 4D, the authors indicate that the SRI-41315 readthrough molecule causes weak readthrough when used alone but it is not visible in the figure probably because the amount of protein loaded is lower, the Western-Blot should be redone by loading the same amount of protein in all lanes.*

Thank you for your comment; we have now repeated the experiment and loaded the same amount of protein in all lanes (Revised Fig. 4D), demonstrating that the SRI-41315 readthrough-inducing molecule leads to nonsense suppression when used alone in the APC R1450X reporter cell line and Colo320 cell line (Fig. 4B and D, respectively).

.

*4*)    *A point which seems essential to me is to show whether the impact on the different steps of translation also makes it possible to increase the efficiency of non-aminoglycoside readthrough compounds (Ataluren, Escin, 2,6-diaminopurine, etc…). These results would make it possible to generalize or not the impact of the different steps of translation on readthrough. The message of the article would then be much stronger.*

Thank you for this comment. We have now addressed this very important point by conducting a series of experiments using non-aminoglycosides readthrough inducers. We used Erythromycin, a macrolide antibiotic that we have previously shown can induce restoration of APC in familial adenomatous polyposis (FAP) patients [15], as well as Escin, a natural herbal product that was shown to induce readthrough [16], and Ataluren (PTC124), a well-documented readthrough inducing agent, which were suggested by the Reviewer.

Indeed, the readthrough effects of these non-aminoglycoside compounds were augmented by affecting the different stages of the protein translation system. This key point is now shown as revised Fig. 5 and has been added to the abstract, results, and discussion sections (Pages 1, 12 and 15). This finding greatly helps generalize the impact of the different translation steps on readthrough and strengthens the article's message.

References:

1.	Catalano T, D'Amico E, Moscatello C, Di Marcantonio MC, Ferrone A, Bologna G, Selvaggi F, Lanuti P, Cotellese R, Curia MC *et al*: **Oxidative Distress Induces Wnt/beta-Catenin Pathway Modulation in Colorectal Cancer Cells: Perspectives on APC Retained Functions**. *Cancers (Basel)* 2021, **13**(23).

2.	Jeong JB, Lee J, Lee SH: **TCF4 Is a Molecular Target of Resveratrol in the Prevention of Colorectal Cancer**. *Int J Mol Sci* 2015, **16**(5):10411-10425.

3.	Morin PJ, Sparks AB, Korinek V, Barker N, Clevers H, Vogelstein B, Kinzler KW: **Activation of beta-catenin-Tcf signaling in colon cancer by mutations in beta-catenin or APC**. *Science* 1997, **275**(5307):1787-1790.

4.	Schneikert J, Grohmann A, Behrens J: **Truncated APC regulates the transcriptional activity of beta-catenin in a cell cycle dependent manner**. *Hum Mol Genet* 2007, **16**(2):199-209.

5.	Yang J, Zhang W, Evans PM, Chen X, He X, Liu C: **Adenomatous polyposis coli (APC) differentially regulates beta-catenin phosphorylation and ubiquitination in colon cancer cells**. *J Biol Chem* 2006, **281**(26):17751-17757.

6.	Munemitsu S, Albert I, Souza B, Rubinfeld B, Polakis P: **Regulation of intracellular beta-catenin levels by the adenomatous polyposis coli (APC) tumor-suppressor protein**. *Proc Natl Acad Sci U S A* 1995, **92**(7):3046-3050.

7.	Kim DY, Kwon YJ, Seo WY, Kim UI, Ahn S, Choi SM, Bang HT, Kim K, Kim JS: **Tankyrase-selective inhibitor STP1002 shows preclinical antitumour efficacy without on-target toxicity in the gastrointestinal tract**. *Eur J Cancer* 2022, **173**:41-51.

8.	Rowan AJ, Lamlum H, Ilyas M, Wheeler J, Straub J, Papadopoulou A, Bicknell D, Bodmer WF, Tomlinson IP: **APC mutations in sporadic colorectal tumors: A mutational "hotspot" and interdependence of the "two hits"**. *Proc Natl Acad Sci U S A* 2000, **97**(7):3352-3357.

9.	Wittenstein A, Caspi M, David Y, Shorer Y, Nadar-Ponniah PT, Rosin-Arbesfeld R: **Serum starvation enhances nonsense mutation readthrough**. *J Mol Med (Berl)* 2019, **97**(12):1695-1710.

10.	Ikediobi ON, Davies H, Bignell G, Edkins S, Stevens C, O'Meara S, Santarius T, Avis T, Barthorpe S, Brackenbury L *et al*: **Mutation analysis of 24 known cancer genes in the NCI-60 cell line set**. *Mol Cancer Ther* 2006, **5**(11):2606-2612.

11.	Wada M, Miller CW, Yokota J, Lee E, Mizoguchi H, Koeffler HP: **Molecular analysis of the adenomatous polyposis coli gene in sarcomas, hematological malignancies and noncolonic, neoplastic tissues**. *J Mol Med (Berl)* 1997, **75**(2):139-144.

12.	Wang B, Wang T, Zhu H, Yan R, Li X, Zhang C, Tao W, Ke X, Hao P, Qu Y: **Neddylation is essential for beta-catenin degradation in Wnt signaling pathway**. *Cell Rep* 2022, **38**(12):110538.

13.	Gao C, Chen G, Kuan SF, Zhang DH, Schlaepfer DD, Hu J: **FAK/PYK2 promotes the Wnt/beta-catenin pathway and intestinal tumorigenesis by phosphorylating GSK3beta**. *eLife* 2015, **4**.

14.	Arab F, Aghaee Bakhtiari SH, Pasdar A, Saburi E: **Evaluation of osteogenic induction potency of miR-27a-3p in adipose tissue-derived human mesenchymal stem cells (AD-hMSCs)**. *Mol Biol Rep* 2023, **50**(2):1281-1291.

15.	Kariv R, Caspi M, Fliss-Isakov N, Shorer Y, Shor Y, Rosner G, Brazowski E, Beer G, Cohen S, Rosin-Arbesfeld R: **Resorting the function of the colorectal cancer gatekeeper adenomatous polyposis coli**. *Int J Cancer* 2020, **146**(4):1064-1074.

16.	Mutyam V, Du M, Xue X, Keeling KM, White EL, Bostwick JR, Rasmussen L, Liu B, Mazur M, Hong JS *et al*: **Discovery of Clinically Approved Agents That Promote Suppression of Cystic Fibrosis Transmembrane Conductance Regulator Nonsense Mutations**. *Am J Respir Crit Care Med* 2016, **194**(9):1092-1103.
